# Recent Advances in *Ent*-Abietane Diterpenes: Natural Sources, Biological Activities and Total Synthesis

**DOI:** 10.3390/molecules31010098

**Published:** 2025-12-25

**Authors:** Lu Li, Yongjie Zhu, Haixia Deng, Liqiong Xie, Chang-Bo Zheng, Jian-Neng Yao, Ji Li

**Affiliations:** 1 School of Pharmaceutical Sciences and Yunnan Key Laboratory of Pharmacology for Natural Products, Kunming Medical University, Kunming 650500, China; 2 Yunnan College of Modern Biomedical Industry, Kunming Medical University, Kunming 650500, China

**Keywords:** *ent*-abietane diterpenoid, isolation, structure elucidation, biological activities, total synthesis, natural products

## Abstract

*Ent*-abietane diterpenoids constitute a class of terpenes with a C_20_ carbon skeleton that underlie a wide range of biological activities. *Ent*-abietane diterpenoids, enantiomeric to the abietane counterparts, represent a family of diterpenoid natural products characterized by their distinct 6/6/6 tricyclic carbocyclic skeletons with exceptional structural complexity. An increasing number of these *ent*-abietane diterpenoids have recently been identified, constituting a well-defined group of naturally occurring compounds. This review provides a comprehensive summary of the natural sources, chemical structures, biological profiles and total synthesis of these *ent*-abietane diterpenoids from 2016 to early 2025.

## 1. Introduction

Diterpenoids constitute a major class of terpenes, characterized by a C_20_ carbon skeleton derived from four isoprene units and remarkable structural diversity that underlies a wide range of biological activities [1,2]. To date, more than 18,000 diterpenoids have been documented and classified into different structural types, taxanes, labdanes, kaurenes and abietanes, to name but a few [3]. The discovery of Taxol (paclitaxel), a landmark anticancer drug derived from taxane family, has spurred tremendous research into diterpenoid chemistry and bioactivity [4,5,6,7].

With the growing number of these diterpenoids identified in recent years, a family of diterpenoid natural products characterized by a unique 6/6/6 tricyclic carbocyclic skeletons and well-orchestrated stereospecificity has emerged and collectively referred to the *ent*-abietane diterpenoids [8,9,10]. The italicized prefix *ent*-, a contracted form of *enantio*-, denotes complete inversion of configuration at all chiral centers (i.e., mirror-image). In this review, compounds possessing additional chiral centers beyond C-5, C-8, C-9, C-10, and C-13 are still classified as *ent*-abietane diterpenoids, provided that their original carbon skeleton is enantiomeric to the standard abietane framework. Indeed, the last decade has witnessed huge progress on the discovery of *ent*-abietane diterpenoids, particularly *ent*-nor-abietanes, dimeric abietanes and rearranged abietanes. Nevertheless, an in-depth and systematic overview of *ent*-abietane diterpenoids, covering their natural sources, biological activities, and total syntheses reported between 2016 and early 2025, remains extremely limited compared with the extensively reviewed abietane diterpenoids [11,12,13,14,15,16,17,18].

Since the naturally occurring compound jolkinolide A was first uncovered in 1972, this group of *ent*-abietane diterpenoids has attracted sustained scientific interest [19]. Notably, a seminal review compiled by Zhang and co-workers summarized *ent*-abietane diterpenoid lactones reported prior to 2016, with particular emphasis on those *ent*-abietane diterpenoids originating from the genus *Euphorbia* [8]. Furthermore, a detailed review dedicated to the representative *ent*-abietane diterpenoid has also been disclosed owing to its potent pharmacological activities [20].

**Figure 1:**
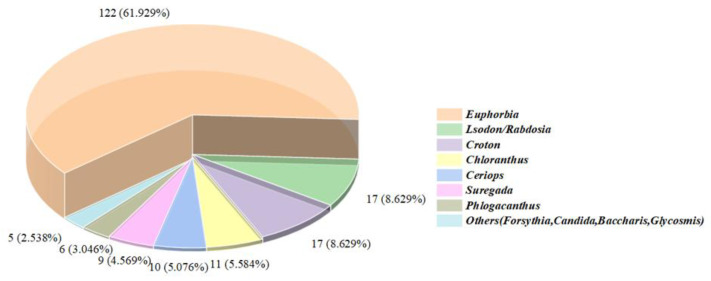
The overview of the number and plant origin of *ent*-abietane diterpenoids.

**Figure 2:**
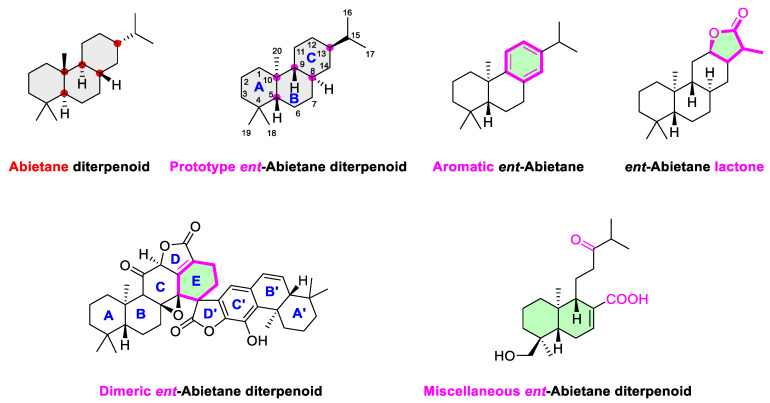
Abietane diterpenoids and five subclass of *ent*-abietane diterpenoids.

Accumulating investigations have revealed an upsurge in the discovery of structurally diverse *ent*-abietane diterpenoids characterized by unprecedented substitution patterns, oxidation levels, and skeletal rearrangements and distribution across many plant genera (Figure 1). To provide an updated and comprehensive overview, this review highlights key advances in 197 *ent*-abietane diterpenoids covering from 2016 to early 2025, encompassing their natural sources, chemical structures, biological profiles and total synthesis. The compounds in this review are categorized into five subclasses (Tables S1–S5), including those prototype, aromatic, lactonized, dimeric and miscellaneous *ent*-abietane diterpenoids (Figure 2).

## 2. Classification of *Ent*-Abietane Diterpenoids

### 2.1. Prototype Ent-Abietane Diterpenoids

Prototype *ent*-abietane diterpenoids are characterized by 6/6/6 tricyclic carbon scaffold and alkyl substitutions at C4, C10 and C13. Typically, compounds of this family commonly bear a dimethyl or oxidized methyl group at C4, an angular methyl group at C10 and an isopropyl or oxidized isopropyl group at C13.

**Figure 3:**
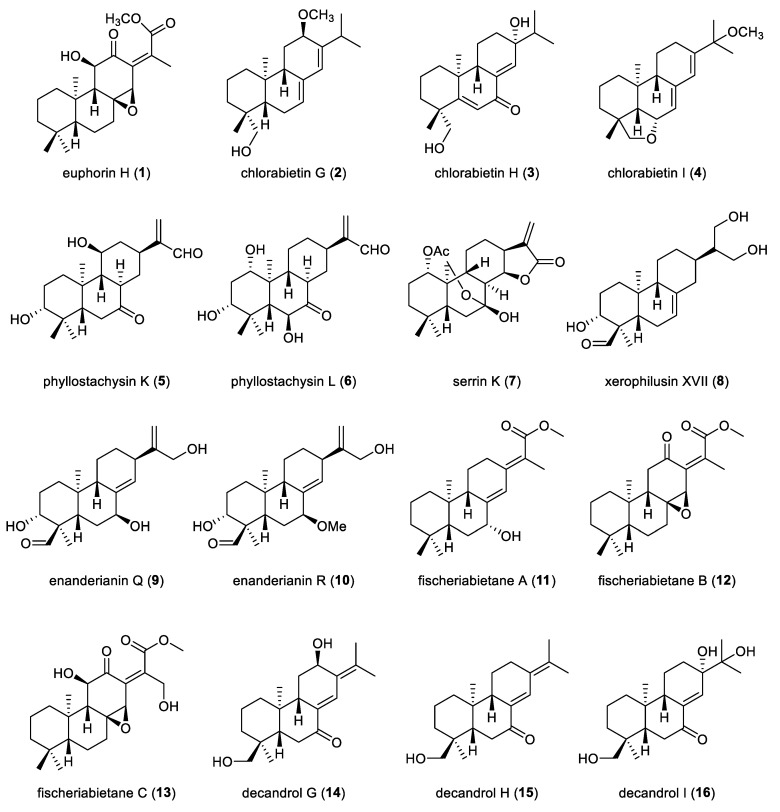
Chemical structures of prototype *ent*-abietane diterpenoids (**1**–**16**).

A prototype *ent*-abietane diterpenoid, euphorin H (**1**), was isolated from the roots of *Euphorbia fischeriana*, a prolific source of polycyclic terpenoids (Figure 3). Compound **1** inhibited the formation of mammospheres in human breast cancer MCF-7 cells at a concentration of 10 µM [21]. A phytochemical investigation of the roots of the rare and endangered plant *Chloranthus oldhamii* led to the isolation and identification of three *ent*-abietane diterpenoids G–I (**2**–**4**). Notably, compound **4** has been recognized as the first example of *ent*-abietane diterpenoid featuring a tetrahydrofuran ring that bridges C-6 and C-19. These three compounds **2**–**4** were evaluated in vitro for their anti-neuroinflammatory activity in LPS-activated murine BV-2 microglial cells. Among them, only compound **2** showed significant inhibition of NO production, with an IC_50_ value of 23.8 µM [22].

Two additional representative prototype *ent*-abietane diterpenoids, phyllostachysins K and L (**5**,**6**), were obtained from the aerial parts of *Isodon phyllostachys*. Detailed NMR analysis revealed that both compounds **5** and **6** share an identical core scaffold bearing an *α*,*β*-unsaturated aldehyde unit at C-13. Compounds **5** and **6** exhibited moderate cytotoxicity against a panel of human tumor cell lines, including HL-60, SMMC-7721, A-549, MCF-**7**, and SW-480, with IC_50_ values ranging from 4.1 to 29.8 μM. Furthermore, both compounds demonstrated potent inhibitory effects on nitric oxide production in LPS-stimulated RAW264.7 macrophages, with IC_50_ values of 1.34 μM and 2.09 μM, respectively [23]. Four diterpenoids, serrin K (**7**), xerophilusin XVII (**8**), and enanderianins Q and R (**9** and **10**), were extracted from the aerial parts of *Isodon serra*. These four compounds (**7**–**10**), featuring an *ent*-abietane skeleton, were identified from this species for the first time. Notably, compound **7** possesses a rare tetrahydropyran ring that links C-20 and C-7, an unusual structural motif within the *ent*-abietane family. Compound **7** exhibited significant inhibition of nitric oxide production in LPS-stimulated RAW264.7 macrophages, with an IC_50_ value of 1.8 μM [24].

Chemical investigation of the roots of *Euphorbia fischeriana* yielded three prototype *ent*-abietane diterpenoids, fischeriabietanes A–C (**11**–**13**). Compounds **12** and **13** are proposed to be biosynthetic precursors of *ent*-abietane diterpenoids featuring an additional five-membered lactone ring. The theoretically calculated ECD spectrum computed for the 5*R*, 7*R*, 9*S*, 10*R* stereoisomer showed excellent agreement with the experimental ECD spectra of compound **11** over the 190–400 nm range, thereby pinpointing the absolute configuration of compound **11** as 5*R*,7*R*,9*S*,10*R*. Furthermore, compounds **12** and **13** displayed moderate antiproliferative effects against Bel-7402 and Panc-28 cancer cell lines. Specifically, compound **12** exhibited Panc-28 cells with an IC_50_ value of 47.2 µM. Compound **13** showed inhibitory activities with IC_50_ values of 12.9 µM and 20.7 µM against the Bel-7402 and Panc-28 cell lines, respectively. These results suggest that the presence of two carbonyl units at C-12 in compounds **12** and **13** may play a crucial role in exerting antitumor activity [25]. Three *ent*-abietanes, decandrols G–I (**14**–**16**), were isolated from the roots of *Ceriops decandra*, an Indian mangrove. Their relative and absolute configurations were determined by HR-ESIMS, extensive 1D and 2D NMR experiments and ECD calculations [26].

**Figure 4:**
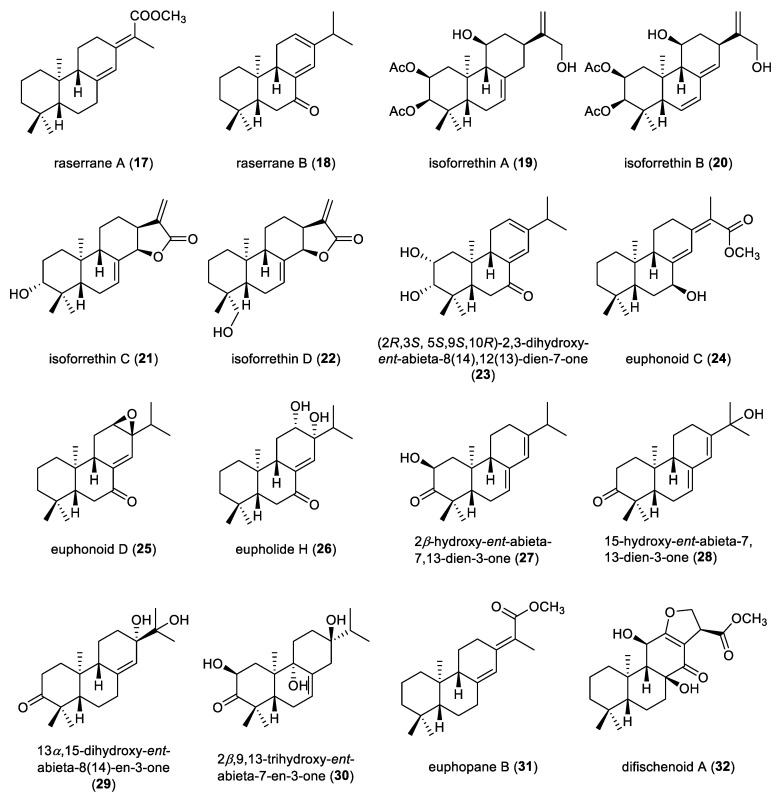
Chemical structures of prototype *ent*-abietane diterpenoids (**17**–**32**).

Raserranes A and B (**17** and **18**) were isolated from the leaves of *Rabdosia serra* (Figure 4). Compound **17** represents a rare example of *ent*-abietane diterpenoids featuring a 16-methoxycarbonyl substituent [27]. Isoforrethins A–D (**19**–**22**) were isolated from the aerial parts of *Isodon forrestii* var. *forrestii.* Compound **19** was subjected to X-ray diffraction analysis, allowing for the assignment of its absolute configuration as 2*S*, 3*R*, 5*S*, 9*S*, 10*R*, 11*S* and 13*R*. Compounds **21** and **22** displayed cytotoxic activities against the SW-80, HL-60, MCF-7 and A-549 cancer cell lines, with IC_50_ values ranging from 10.1 to 20.2 µM [28]. An *ent*-abietane diterpenoid (**23**) was isolated from the aerial parts of *Euphorbia thymifolia*. Its structure and relative configuration were elucidated through extensive spectroscopic analysis. The absolute configuration of compound **23** was established by comparing its experimental ECD spectrum with that calculated using time-dependent density functional theory (TDDFT) at the B3LYP/6–311+G(d,p) level. The experimental ECD spectrum of compound **23** closely matched with the calculated ECD spectrum for the (2*R*,3*S*,5*S*,9*S*,10*R*) stereoisomer. Accordingly, compound **23** was assigned as (2*R*,3*S*,5*S*,9*S*,10*R*)-2,3-dihydroxy-*ent*-abieta-8(14),12(13)-dien-7-one [29].

Euphonoids C and D (**24**,**25**) were extracted from the roots of *Euphorbia fischeriana*. The absolute configurations of both compounds were confirmed via ECD calculations [30]. Eupholide H (**26**), a representative *ent*-abietane diterpenoid, was also obtained from the roots of *E. fischeriana*. The absolute configuration of compound **26** was elucidated by ECD calculations. Compound **26** showed moderately inhibitory activity against the proliferation of *Mycobacterium tuberculosis* H37Ra, with a MIC value of 50 μM [31]. Phytochemical investigation of the leaves of *Croton mubango*, collected in the Democratic Republic of the Congo, led to the isolation of four *ent*-abietane diterpenoids, 2*β*-hydroxy-*ent*-abieta-7,13-dien-3-one (**27**), 15-hydroxy-*ent*-abieta-7,13-dien-3-one (**28**), 13*α*,15-dihydroxy-*ent*-abieta-8(14)-en-3-one (**29**), and 2*β*,9,13-trihydroxy-*ent*-abieta-7-en-3-one (**30**) [32,33]. A plant-derived *ent*-abietane diterpenoid, euphopane B (**31**) was isolated from the roots of *Euphorbia pekinensis*. Its absolute configuration was rationalized by ECD calculations [34]. Compound **31** exhibited moderate cytotoxicity against human prostate cancer C4-2B cells with an IC_50_ value of 16.9 µM. A highly oxidized *ent*-abietane diterpenoid, difischenoid A (**32**), was obtained from the roots of wild *Euphorbia fischeriana*. The absolute configuration of compound **32** was unambiguously established by single-crystal X-ray diffraction analysis. Moreover, compound **32** showed cytotoxicity against Hela cells, with an IC_50_ value of 15.47 µM [35].

**Figure 5:**
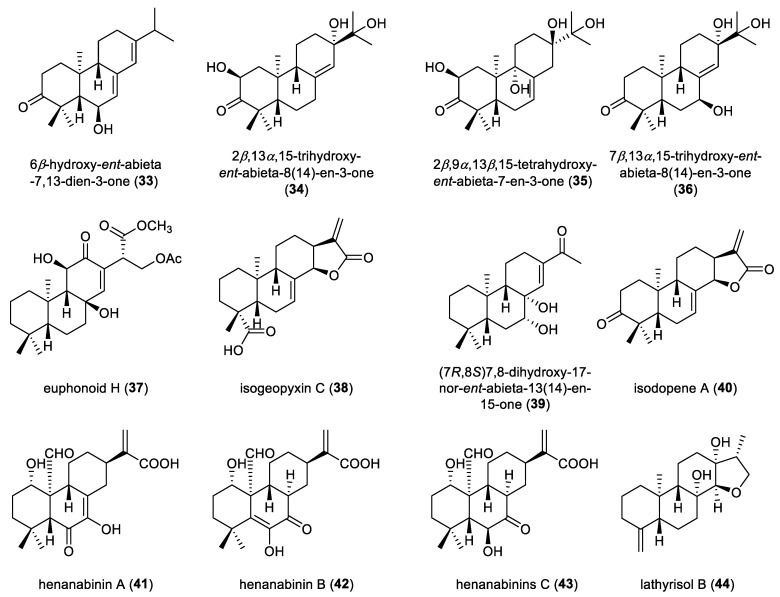
Chemical structures of prototype *ent*-abietane diterpenoids (**33**–**44**).

Three *ent*-abietane diterpenoids were isolated from the leaves of *Croton cascarilloide* and identified as 6*β*-hydroxy-*ent*-abieta-7,13-dien-3-one (**33**), 2*β*,13*α*,15-trihydroxy-*ent*-abieta-8(14)-en-3-one (**34**), and 2*β*,9*α*,13*β*,15-tetrahydroxy-*ent*-abieta-7-en-3-one (**35**) (Figure 5). These three compounds showed weak antimicrobial activities against the Gram-positive bacteria T25-17, C159-6 and sp.8152, with MIC values below 50 μg/mL [36]. An *ent*-abietane diterpenoid, namely 7*β*,13*α*,15-trihydroxy-*ent*-abieta-8(14)-en-3-one (**36**), was also extracted from the leaves of *Croton lachnocarpus* [37]. Phytochemical investigation of *Euphorbia fischeriana* resulted in the isolation of an *ent*-abietane diterpenoid, euphonoid H (**37**). Its absolute configuration was determined by ECD calculations. Compound **37** showed significant antiproliferative activity against the human prostate cancer cell lines C4-2B and C4-2B/ENZR, with IC_50_ values of 5.52 and 4.16 µM, respectively [38]. Isogeopyxin C (**38**) was identified from the fermentation broth of *Geopyxis* sp. XY93, an endophytic fungal strain inhibiting *Isodon parvifolia*. Its structural elucidation was unequivocally achieved by single-crystal X-ray diffraction analysis [39].

An *ent*-norabietane diterpenoid (**39**) was isolated from the rhizomes of *Euphorbia jolkinii*. Its chemical structure was identified through analysis of NMR data combined with ECD calculations, and it was assigned as (7*R*,8*S*)-7,8-dihydroxy-17-nor-*ent*-abieta13(14)-en-15-one [40]. Isodopene A (**40**), an *ent*-abietane diterpenoid isolated from the roots of *Isodon ternifolius*, showed strong inhibitory activity against DNA topoisomerase IB (TOP1) [41]. Three *ent*-abietane diterpenoids, henanabinins A–C (**41**–**43**), were extracted from the aerial parts of *Isodon rubescens*. The absolute configuration of compound **41** was established by single-crystal X-ray diffraction analysis [42]. An *ent*-norabietane diterpenoid featuring an exocyclic olefin at C-4, lathyrisol B (44), was isolated from the roots of *Euphorbia lathyrism*. Its absolute configuration was established by single-crystal X-ray diffraction analysis. Compound **44** enhanced the expression of C/EBP homologous protein (CHOP) in MIA PaCa-2 human pancreatic cancer cells, an effect consistent with that of its congener lathyrisol A [43].

### 2.2. Aromatic Ent-Abietane Diterpenoids

Aromatic *ent*-abietane diterpenoids are defined as a class of C-ring aromatized *ent*-abietane diterpenoids and C-ring aromatized *ent*-norabietane diterpeniods, which are naturally occurring carbon-reduced derivatives of *ent*-abietane diterpenoids.

**Figure 6:**
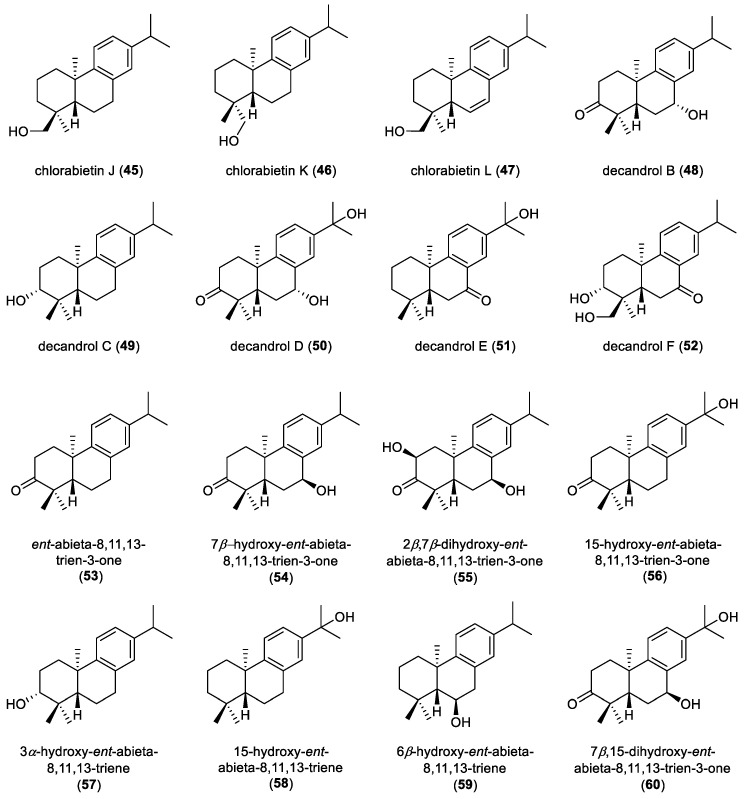
Chemical structures of aromatic *ent*-abietane diterpenoids (**45**–**60**).

Chlorabietins J–L (**45**–**47**), three aromatic *ent*-abietane diterpenoids isolated from the roots of *Chloranthus oldhamii*, were obtained as naturally occurring constituents (Figure 6). These metabolites have been proposed as a new class of chemotaxonomic marker for the genus *Chloranthus* [22]. Five aromatic *ent*-abietanes, decandrols B–F (**48**–**52**), were isolated from the roots of *Ceriops decandra*, an Indian mangrove collected in the swamp of Godavari estuary, Andhra Pradesh. Their absolute configurations were unequivocally deduced by ECD calculations. Among them, compound **49** and **51** showed NF-κB inhibitory activity at a concentration of 100 μM [26].

**Figure 7:**
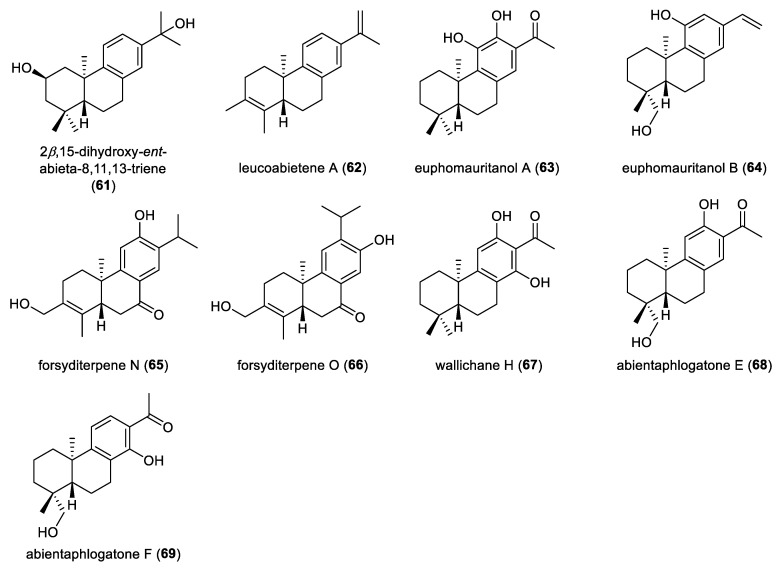
Chemical structures of aromatic *ent*-abietane diterpenoids (**61**–**69**).

Seven aromatic *ent*-abietane diterpenoids were identified from the leaves of *Croton mubango* and characterized as *ent*-abieta-8,11,13-trien-3-one (**53**), 7*β*-hydroxy-*ent*-abieta-8,11,13-trien-3-one (**54**), 2*β*,7*β*-dihydroxy-*ent*-abieta-8,11,13-trien-3-one (**55**), 15-hydroxy-*ent*-abieta-8,11,13-trien-3-one (**56**), 3*α*-hydroxy-*ent*-abieta-8,11,13-triene (**57**), 15-hydroxy-*ent*-abieta-8,11,13-triene (**58**), and 6*β*-hydroxy-*ent*-abieta-8,11,13-triene (**59**) [32]. Phytochemical investigation of the leaves of *Croton lachnocarpus* resulted in the identification of two aromatic *ent*-abietane diterpenoids, 7*β*,15-dihydroxy-*ent*-abieta-8,11,13-trien-3-one (**60**) and 2*β*,15-dihydroxy-*ent*-abieta-8,11,13-triene (**61**) [37]. Leucoabietene A (**62**), a rearranged *ent*-abietane diterpenoid characterized by an aromatic C ring, was isolated from the non-polar fraction of the leaves of *Leucosceptrum canum*, a large woody plant to produce sesterterpenoids as its major chemical constituents (Figure 7). Compound **62** effectively reversed fluconazole resistance in fluconazole-resistant *Candida albicans*. When the concentration of compound **62** exceeded 32 µg/mL, the antifungal efficacy of fluconazole was restored, yielding inhibition rates greater than 88%. These findings indicate that compound **62** may function as a potent chemosensitizer capable of overcoming fluconazole resistance [44].

Chemical investigation of the monoecious succulent shrub *Euphorbia mauritanica* led to isolation and identification of two aromatic *ent*-norabietane diterpenoids, euphomauritanols A and B (**63** and **64**). The absolute configurations of these two compounds (**63** and **64**) were deduced as 5*R*, 10*S* by employing TDDFT-ECD calculations. Both compounds exhibited antiproliferative activities against murine melanoma B16-BL6 cell lines, with IC_50_ values of 10.28 μM and 20.22 μM, respectively. Furthermore, molecular docking within the active sites of BRAF^V600E^ and MEK1 kinases provided a structural rational for their inhibitory effects. In addition, the in silico pharmacokinetic profiling using SwissADME indicated that both compounds possessed favorable drug-like properties and oral bioavailability [45]. From the seeds of *Forsythia suspensa*, two rearranged *ent*-abietane diterpenoids, forsyditerpenes N and O (**65** and **66**), were obtained. Wallichane H (**67**) was isolated from the whole plant of *Euphorbia wallichii* [46].

The isolation and characterization of two aromatic *ent*-norabietane diterpenoids, abientaphlogatones E and F (**68** and **69**), from the aerial parts of *Phlogacanthus curviflorus*, was uncovered. The absolute configurations of these two compounds were rationalized by ECD calculations [47]. Notably, the discovery of compounds **68** and **69** represents the first report of *ent*-norabietane diterpenoids in the genus *Phlogacanthus.* Compound **69** showed neuroprotective activity in PC12 cell injury models induced by H_2_O_2_ and MPP^+^, underscoring the importance of the hydroxyl substituents on the aromatic C-ring for bioactivity.

### 2.3. Ent-Abietane Diterpenoid Lactones

*Ent*-abietane diterpenoid lactones are collectively referred to those abietane diterpenoids, where the ring D harbors *γ*-butenolide, *γ*-butyrolactone or their derivatives.

**Figure 8:**
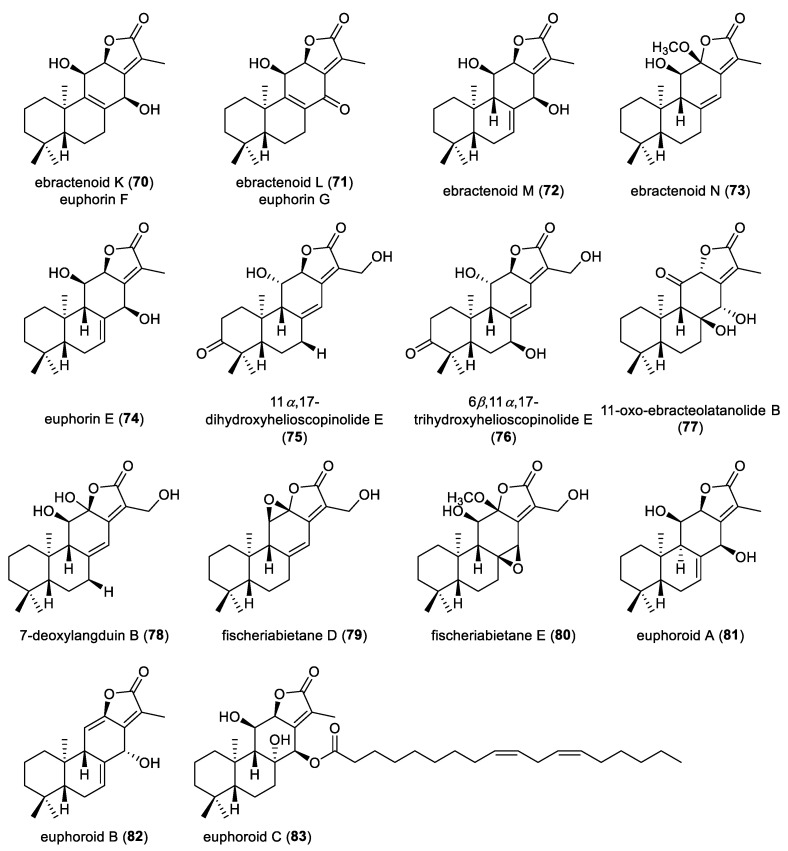
Chemical structures of *ent*-abietane diterpenoid lactones (**70**–**83**).

Phytochemical exploration of *Euphorbia ebracteolata* has expanded the structural diversity of *ent*-abietane diterpenoids, leading to the isolation of four metabolites, ebractenoids K–N (**70**–**73**) (Figure 8). Notably, ebractenoids K and L were also reported as euphrins F and G, respectively. Compounds **71** and **73** showed potent anti-inflammatory effects by markedly suppressing LPS-induced nitric oxide production in RAW264.7 macrophages with IC_50_ values of 0.69 µM and 1.97 µM, respectively [48]. From the roots of *Euphorbia fischeriana*, an *ent*-abietane diterpenoid, euphorin E (**74**), was isolated and exhibited the formation of mammospheres in human breast cancer MCF-7 cells at a concentration of 10 µM [21]. Additional investigation of the same plant afforded four *ent*-abietane diterpenoid lactones, 11*α*,17-dihydroxyhelioscopinolide E (**75**), 6*β*,11*α*,17-trihydroxyhelioscopinolide E (**76**), 11-*oxo*-ebracteolatanolide B (**77**), and 7-deoxylangduin B (**78**). Their absolute configurations were identified by TDDFT-based ECD calculations [49]. Further investigation on the roots of *Euphorbia fischeriana* yielded two *ent*-abietane diterpenoid lactones, fischeriabietanes D and E (**79** and **80**). The absolute configurations of compound **79** were determined by ECD calculations [25].

**Figure 9:**
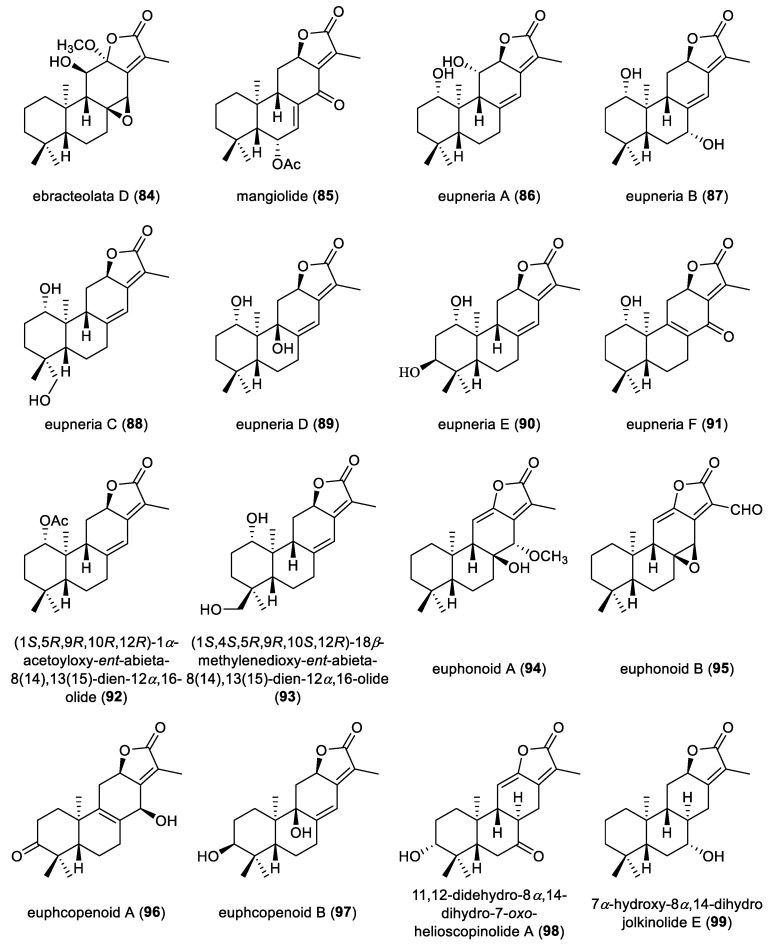
Chemical structures of *ent*-abietane diterpenoid lactones (**84**–**99**).

Euphoroids A–C (**81**–**83**), bearing a distinctive lactone moiety, were extracted from the roots of *Euphorbia ebracteolate*. Chemical degradation of compound **83**, followed by HPLC analysis, revealed the presence of a linoleic acid residue in its structure. These three compounds were subjected to cytotoxic evaluation against several human cancer cells. The compound **83** showed antiproliferation of four tested human cancer cells A549, MCF-7, Lovo and SH-SY5Y, with IC_50_ values below 30 µM [50]. Ebracteolata D (**84**) was obtained as an *ent*-abietane diterpenoid from the roots of *Euphorbia ebracteolate* (Figure 9) [51]. An *ent*-abietane diterpenoid lactone, mangiolide (**85**), was isolated from the stem bark of *Suregada zanzibariensis* via anticancer bioassay-guided fractionation. Compound **85** showed pronounced cytotoxicity against TK10 renal, UACC62 melanoma and MCF7 breast cancer cell lines, with total growth inhibition (TGI) values of 0.20, 0.16 and 0.89 μM and growth inhibition (GI_50_) values of 54, 80 and 134 nM, respectively [52]. Chemical investigation of *Euphorbia neriifolia* yielded six *ent*-abietane diterpenoid lactones, eupnerias A–F (**86**–**91**). In the follow-up anti-inflammatory and anti-influenza virus bioassay evaluation, none of them exhibited significant activity under the tested conditions [53].

Two *ent*-abietane-type diterpenoids, specifically (1*S*,5*R*,9*R*,10*R*,12*R*)-1*α*-acetoyloxy-*ent*-abieta-8(14),13(15)-dien-12a,16-olide (**92**) and (1*S*,4*S*,5*R*,9*R*,10*S*,12*R*)-18*β*-methylenedioxy-*ent*-abieta-8(14),13(15)-dien-12*α*,16-olide (**93**), were isolated from the methanolic extract of *Euphorbia royleana*. Their absolute configurations of these two compounds were determined by ECD calculations. Compound **92** displayed strong inhibitory activity on nitric oxide production in LPS-stimulated BV-2 cells (IC_50_ = 12.0 μM) and molecular docking studies suggested that its anti-inflammatory activity may arise from interactions within the catalytic pocket of inducible nitric oxide synthase (iNOS) [54]. Euphonoids A and B (**94,95**) were isolated from the roots of *Euphorbia fischeriana*. X-ray crystallography analysis of compound **94** pinpointed its absolute configurations. Compound **94** exhibited significant antiproliferative activity against the human prostate cancer cell lines C4-2B and C4-2B/ENZR with IC_50_ values of 9.18 and 9.7 µM, respectively. Compounds **95** also displayed comparably antiproliferative activity in a parallel assay (IC_50_ = 13.4 and 11.1 µM) [30].

Two *ent*-abietane diterpenoid lactones, euphcopenoids A and B (**96** and **97**), were isolated from the whole plant of *Euphorbia helioscopia*. Their absolute configurations were deduced via ECD calculations [55]. Two *ent*-abietane diterpenoid lactones, 11,12-didehydro-8*α*,14-dihydro-7-oxo-helioscopinolide A (**98**) and 7*α*-hydroxy-8*α*,14-dihydro jolkinolide E (99), were isolated from the whole plant of *Euphorbia peplus*. Both compounds **98** and **99** were inactive at a concentration of 40 μM in the cytotoxicity assays against a panel of five human tumor cell lines [56].

**Figure 10:**
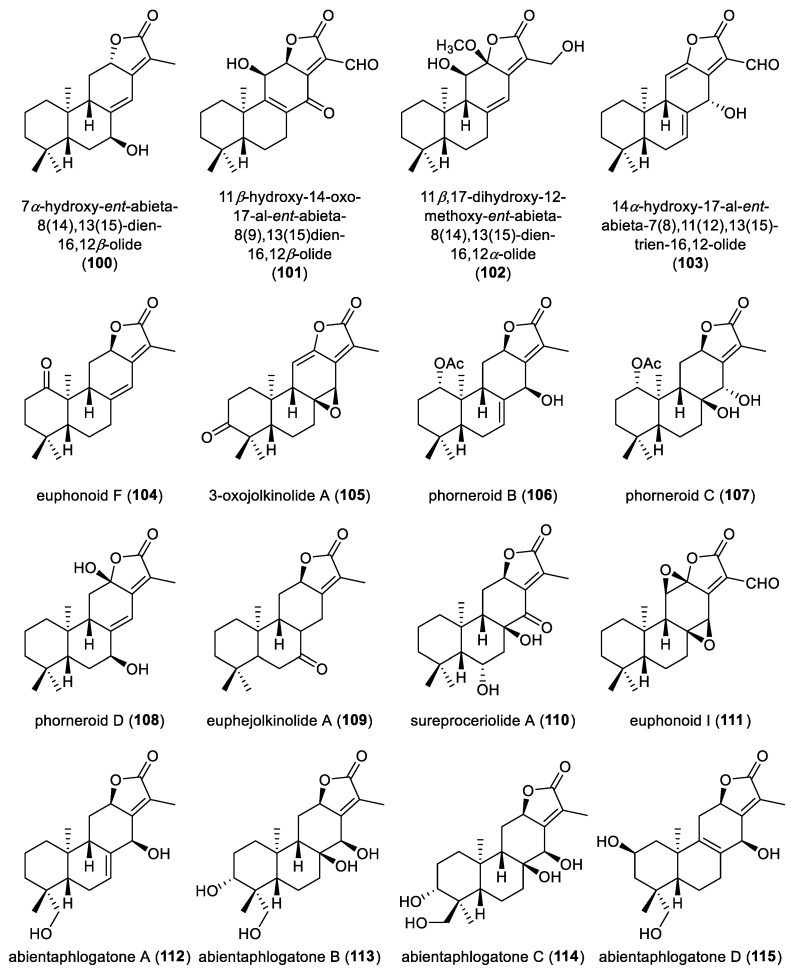
Chemical structures of *ent*-abietane diterpenoid lactones (**100**–**115**).

From the aerial parts of *Baccharis sphenophylla*, the hexane extract furnished 7*α*-hydroxy-*ent*-abieta-8(14),13(15)-dien-16,12*β*-olide (**100**) (Figure 10). Compound **100** exhibited moderate antiproliferative activity against NTCT cells, with an EC_50_ value of 21.3 μM. While this compound showed low toxicity towards NCTC cells, with a CC_50_ value exceeding 200 μM, resulting in a selectivity index (SI) value exceeding 9.4 [57]. Chemical investigation of the 95% ethanol extract the roots of *Euphorbia wallichii* yielded three *ent*-abietane diterpenoid lactones, 11*β*-hydroxy-14-oxo-17-al-*ent*-abieta-8(9),13(15)dien-16,12*β*-olide (**101**), 11*β*,17-dihydroxy-12-methoxy-*ent*-abieta-8(14),13(15)-dien-16,12*α*-olide (**102**), and 14*α*-hydroxy-17-al-*ent*-abieta-7(8),11(12),13(15)-trien-16,12-olide (**103**). These three isolates **101**–**103** were evaluated for their antimicrobial activities *in vitro* against six pathogenic microorganisms and exhibited weak inhibition of the tested Gram-positive strains T25-17, C159-6 and sp.8152, with MIC values below 60 μg/mL [58]. An *ent*-abietane diterpenoid lactone, euphonoid F (**104**), was isolated from the aerial parts of *Euphorbia antiquorum* [59].

From *Glycosmis pentaphylla*, 3-oxojolkinolide A (**105**) was obtained as the first *ent*-abietane diterpenoid lactone reported from genus *Glycosmis* [60]. Three *ent*-abietane diterpenoid lactones, phorneroids B–D (**106**–**108**), were obtained from the aerial parts of *Euphorbia neriifolia*. The absolute configuration of compound **108** was established through a combination of ECD calculations and single-crystal X-ray crystallographic analysis. Compounds **106** and **107** displayed moderate cytotoxicities against A549 and HL-60 tumor cell lines, with IC_50_ values ranging from 2.5 to 9.0 μM, with adriamycin as the positive control, while compound **108** lacked detectable activity [61].

Euphejolkinolide A (**109**), an *ent*-abietane lactone, was isolated from the whole plant of *Euphorbia peplus* with its absolute configuration unambiguously confirmed by single-crystal X-ray diffraction analysis. Biological evaluation revealed that compound **109** induced lysosome biogenesis and autophagy via activating the translocate of transcription factor EB (TFEB). The structure-activity relationship (SAR) analysis indicates that the carbonyl group at C-7 in compound **109** is crucial for maintaining this activity [62]. Phytochemical investigation of the leaves and roots of *Suregada procera* led to the identification of an *ent*-abietane diterpenoid lactone, sureproceriolide A (**110**). DFT-based ECD calculations pinpointed its absolute configuration. Compound **110** showed modest antibacterial activity against the Gram-positive bacterium *Staphylococcus lugdunensis* strain, with a MIC value of 31.44 μM [63]. Chemical investigation of the roots of *Euphorbia fischeriana* led to the identification of an *ent*-abietane diterpenoid lactone, euphonoid I (**111**). Its absolute configuration was assigned via ECD calculations. Compound **111** showed remarkable antiproliferative activity against the human prostate cancer cell lines C4-2B and C4-2B/ENZR, with IC_50_ values of 4.49 and 5.74 μM [38].

**Figure 11:**
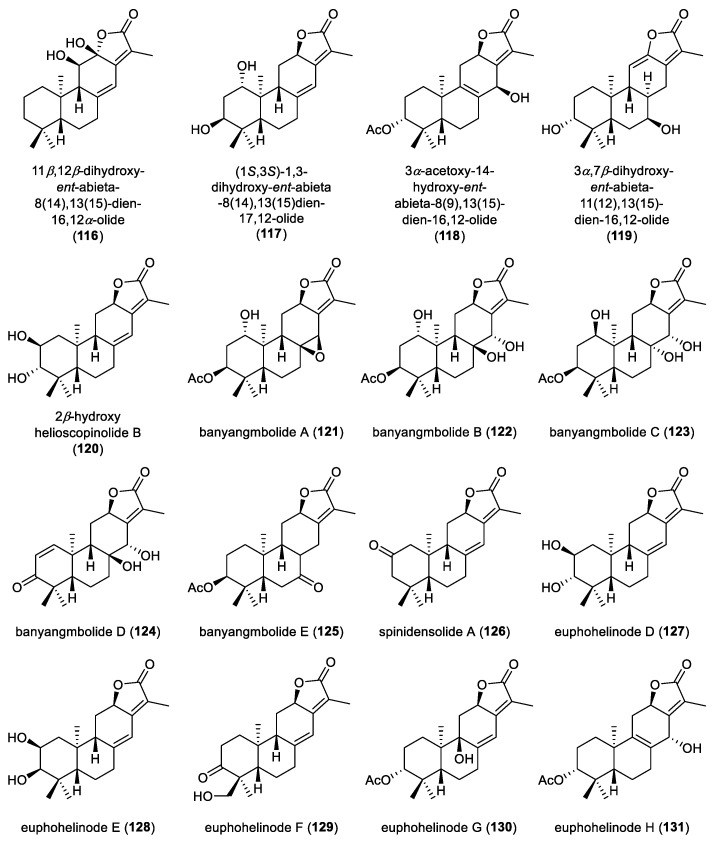
Chemical structures of *ent*-abietane diterpenoid lactones (**116**–**131**).

Abientaphlogatones A−D (**112**–**115**), possessing an *ent*-abietane scaffold, were isolated from the aerial parts of *Phlogacanthus curviflorus*. Their absolute configurations were established by ECD calculations. In the *β*-hematin formation inhibition assay, compounds **113** and **115** exhibited antimalarial activities, with IC_50_ values of 22.85 and 14.21 μM, respectively. Moreover, compound **115** could significantly alleviate H_2_O_2_-induced injury in PC12 cells at concentrations of 20 and 50 μmol·L^−1^ [47]. Phytochemical investigation of the roots of *Euphorbia phosphorea* utilizing chromatographic separation led to the isolation of an *ent*-abietane diterpenoid lactone, identified as 11*β*,12*β*-dihydroxy-*ent*-abieta-8(14),13(15)-dien-16,12*α*-olide (**116**) (Figure 11). An *ent*-abietane diterpenoid lactone, (1*S*,3*S*)-1,3-dihydroxy-*ent*-abieta-8(14),13(15)dien-17,12-olide (**117**), was isolated from the dried roots of *Euphorbia jolkinii.* The extract of *Euphorbia fischeriana* yielded three *ent*-abietane diterpenoid lactones, namely 3*α*-acetoxy-14-hydroxy-*ent*-abieta-8(9),13(15)-dien-16,12-olide (**118**), 3*α*,7*β*-dihydroxy-*ent*-abieta-11(12),13(15)-dien-16,12-olide (**119**), and 2*β*-hydroxy helioscopinolide B (**120**). Among them, compound **118** exhibited moderate cytotoxic activity against the HL-60, SMMC-7721 cell lines with IC_50_ values of 15.3 and 29.0 μM, respectively [64]. Phytochemical analysis of an herbarium specimen of *Suregada occidentalis* led to the identification of five *ent*-abietane diterpenoids featuring *α*-methyl-*α*,*β*-unsaturated-*γ*-lactone moiety, designated as banyangmbolides A–E (**121**–**125**) [65].

**Figure 12:**
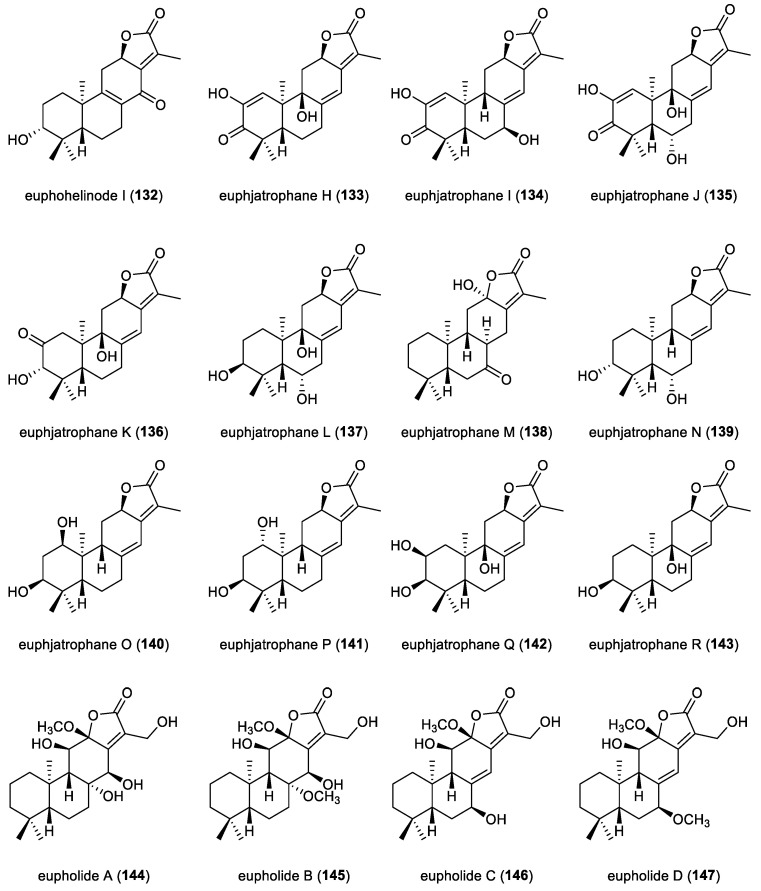
Chemical structures of *ent*-abietane diterpenoid lactones (**132**–**147**).

Spinidensolide A (**126**), an *ent*-abietane lactone isolated from the roots of *Euphorbia spinidens*, exhibited negligible antimicrobial activity [66]. Six *ent*-abietane lactones, euphohelinodes D–I (**127**–**132**), were obtained from *Euphorbia helioscopia* via bioassay-guided fractionation (Figure 12). Their absolute configurations were determined by ECD calculations and the structures of compounds **130** and **131** were further confirmed by single-crystal X-ray diffraction analysis. Notably, compound **130** represents a rare *ent*-abietane diterpenoid characterized by a *β*-oriented hydroxyl group at C9. Compound **131** inhibited NO production in LPS-induced RAW264.7 macrophages, with an IC_50_ value of 30.23 μM. Mechanistic studies suggested that its anti-inflammatory activity was mediated through inhibition of the NF-κB signaling pathway and the downregulation of proinflammatory mediators COX-2 and iNOS [67].

**Figure 13:**
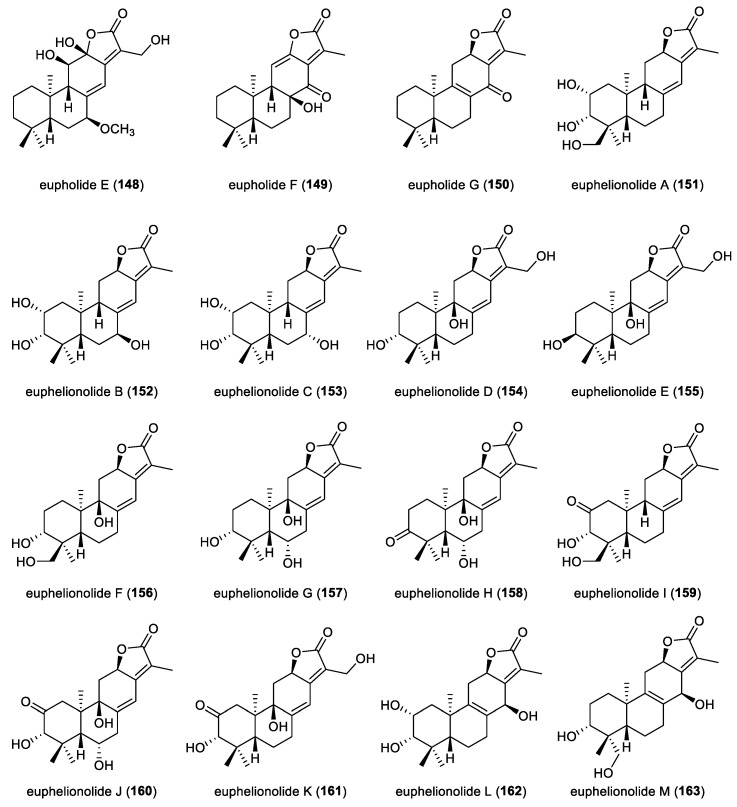
Chemical structures of *ent*-abietane diterpenoid lactones (**148**–**163**).

A comprehensive phytochemical investigation of the whole plant of *Euphorbia peplus* led to the isolation of eleven *ent*-abietane diterpenoid lactones, euphjatrophanes H–R (**133**–**143**). The absolute configurations of compounds **133** and **134** and **136**–**139** were unequivocally established by single-crystal X-ray diffraction analysis. In anti-inflammatory assays using RAW264.7 macrophages, compounds **138**–**141** significantly inhibited nitric oxide production. Specifically, at a concentration of 10 μM, compounds **138**, **141** and **143** markedly downregulated the mRNA expression of IL-6, IL-1*β*, and TNF-*α* in LPS-induced RAW264.7 macrophages. Compound **138** showed a dose-dependent inhibition of these proinflammatory mediators, effectively attenuated FOXO1 expression and reduced NF-κB p65 phosphorylation. Collectively, these results suggested that compound **138** is a promising lead candidate for the development of therapeutics targeting inflammation-related diseases [68]. Seven *ent*-abietane diterpenoid lactones, eupholides A−G (**144**–**150**), were isolated from the roots of *Euphorbia fischeriana* (Figure 13). The absolute configurations of compounds **144** and **149** were established by single-crystal X-ray diffraction analysis. In biological evaluation, compounds **149** and **150** showed moderate inhibition of *Mycobacterium tuberculosis* H37Ra with a MIC value of 50 μM. Moreover, compound **150** demonstrated potent inhibition of human carboxylesterase 2 (HCE 2), with an IC_50_ value of 7.3 nM, highlighting it as a metabolically relevant and pharmacologically significant molecule [31].

**Figure 14:**
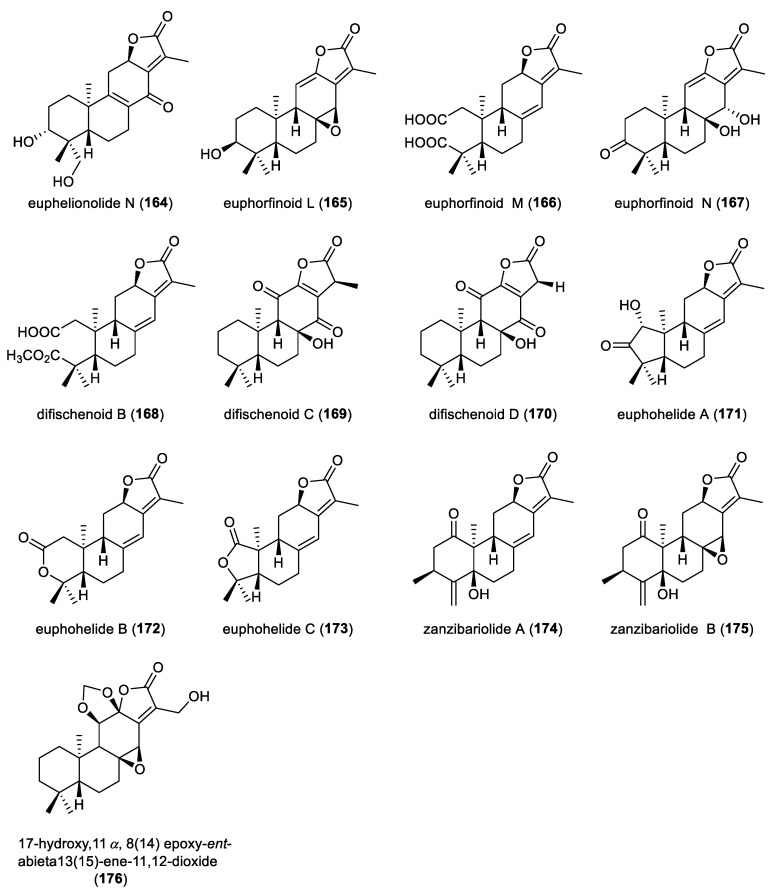
Chemical structures of *ent*-abietane diterpenoid lactones (**164**–**176**).

A comprehensive phytochemical investigation of the aerial parts of *Euphorbia helioscopia* resulted in the isolation of fourteen highly oxygenated *ent*-abietane diterpenoid lactones, euphelionolides A–N (**151**–**164**) (Figure 14). Compounds **156** and **164** displayed notable cytotoxicities against MCF-7 and PANC-1 cancer cell lines, with IC_50_ values ranging from 9.5 to 10.7 μM [69]. Euphorfinoid L (**165**) was isolated from the roots of the wild *Euphorbia fischeriana*. Compound **165** was structurally elucidated by using NMR, MS and ECD analysis. Compound **165** exhibited weak inhibitory activity against acetylcholinesterase (AChE), with an IC_50_ value of 147.51 μM [70]. Two *ent*-abietane diterpenoids, euphorfinoids M and N (**166** and **167**), were extracted from the roots of wild *Euphorbia fischeriana*. Notably, compound **166** represents a rare example of ring A-seco *ent*-abietane diterpenoid lactone. Compound **167** showed antiproliferative activity against Hela cell lines, with an IC_50_ value of 3.62 μM [71]. Three *ent*-abietane diterpenoids, difischenoids B–D (168–170), were isolated from the roots of wild *Euphorbia fischeriana*. Compound **168** was a 17-nor-*ent*-abietane diterpenoid. The absolute configurations of compounds **168**–**170** were established by ECD calculations. Compound **168** showed cytotoxic activity against Hela cell lines, with an IC_50_ value of 3.75 μM. Mechanistic studies revealed that apoptosis induced by compound **169** was associated with increased reactive oxygen species (ROS), enhanced Ca^2+^ influx, and dissipation of the mitochondrial membrane potential [35].

Three *ent*-abietane norditerpenoid lactones, euphohelides A−C (**171**–**173**), were isolated from the whole plant of *Euphorbia helioscopia*. Compound **171** features a distinctive 5/6/6/5 tetracyclic *ent*-norabietane skeleton, whereas compounds **172** and **173** harbor dilactone frameworks. Single-crystal X-ray crystallography analysis pinpointed the chemical structure of compound **171**, which was also semi-synthesized in 4 steps from a plausible precursor 2*α*-hydroxyhelioscopinolide B. Compound **171** inhibited LPS-induced nitric oxide production in RAW264.7 macrophages (IC_50_ value of 32.98 μM), suggesting that its anti-inflammatory effect may involve the modulation of the NF-κB signaling pathway [72].

Chemical investigation on the leaves of *Suregada zanzibariensis* led to the identification of two rearranged *ent*-abietane diterpenoid lactones possessing a terminal double band, zanzibariolides A and B (**174** and **175**). Their absolute configurations were established by single-crystal X-ray diffraction analysis. Both compounds **174** and **175** were screened for their anti-herpes simplex virus type 2 (HSV-2) activities, but showed negligible antiviral effects [73]. An *ent*-abietane diterpenoid (**176**) was isolated from the roots of *Euphorbia fischeriana*. The absolute configuration of compound **176** was established by ECD calculations and identified as 5*R*, 8*S*, 9*R*, 10*R*, 12*R*, 14*R*. Thus compound **176** was named as 17-hydroxy,11*α*, 8(14) epoxy-*ent*-abieta-13(15)-ene-11,12-dioxide [74].

### 2.4. Dimeric Ent-Abietane Diterpenoids

Dimeric *ent*-abietane diterpenoids, also referred to *ent*-abietane bisditerpenoids, constitute a structurally diverse class of natural products distinguished by their varied linkage patterns [75,76,77]. Homodimers arise from the coupling of two identical monomeric units sharing the same carbon skeleton, whereas heterodimers result from the fusion of two distinct diterpenoid frameworks. These dimeric architectures often display remarkable molecular complexity and exhibit enhanced or unique biological profiles compared with their monomeric precursors [78,79,80,81,82].

**Figure 15:**
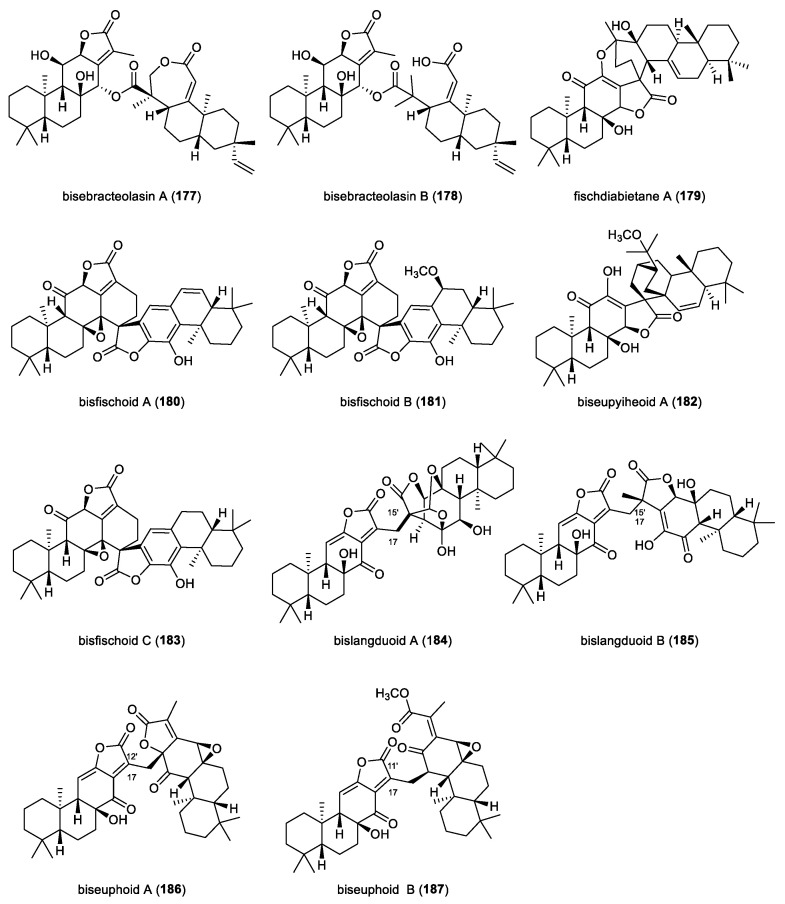
Chemical structures of dimeric *ent*-abietane diterpenoids (**177**–**187**).

Two oxygen-bridged heterodimeric diterpenoids incorporating a monomer unit with a rare *seco*-rosane scaffold, bisebracteolasins A and B (**177** and **178**), were identified from the roots of *Euphorbia ebracteolate* (Figure 15). The absolute configuration of compound **177** was determined by single-crystal X-ray diffraction analysis. Both compound **177** and **178** displayed antiproliferative activities against five human cell lines HL-60, A549, SMMC-7721, MCF and SW-480, with IC_50_ values ranging from 2.61 to 14.09 μM. Importantly, flow cytometry analysis revealed that compound **177** induced apoptosis in SMMC-7721 cells at a concentration of 20 μM, while compound **178** did not. Notably, both dimers suppressed the growth of the CD44^+^ colorectal cancer stem cell line P6C, with IC_50_ values of 16.48 and 34.76 μM, respectively. Preliminary biological assays further revealed that compound **178** inhibited tumoursphere formation and impaired P6C migration, demonstrating its as lead compound for targeting cancer stem cell-driven tumor progression and metastasis [83].

Fischdiabietane A (**179**), an *ent*-abietane dimer endowed with an unprecedented 6/6/6/5/7/6/6/6/ ring framework, was isolated from the roots of *Euphorbia fischeriana.* Its structure was unambiguously established by X-ray diffraction analysis. Compound **179** represents the first *ent*-abietane dimer proposed to arise biosynthetically through a Diels-Alder cycloaddition. Compound **179** exhibited potent cytotoxicity toward the T47D breast cancer line, with an IC_50_ value of 6.51 μM, displaying nearly sixfold greater potency than the positive control cisplatin. Mechanistic studies revealed that compound **179** induced apoptosis in T47D cells via caspase-3 activation and poly(ADP-ribose) polymerase (PARP) degradation [84].

Two *ent*-abietane dimers putatively formed by an intermolecular [4 + 2] cycloaddition, bisfischoids A and B (**180**,**181**), were isolated from *Euphorbia fischeriana*. Both compounds **180** and **181** inhibited soluble epoxide hydrolase (sEH), with IC_50_ values of 9.9 and 10.29 μM, respectively, suggesting therapeutic potential for inflammation-related disorders. Molecular docking and molecular dynamic simulation indicated that interactions with the catalytic cavity, particularly the amino acid residue Tyr343, play a key role in their inhibitory properties of sEH [85].

Biseupyiheoid A and bisfischoid C (**182** and **183**) representing two unprecedented *ent*-abietane dimers, also derived from *Euphorbia fischeriana*. Compound **182** possesses a rare spirocyclic 6/6/6/5/6/6/6/6 framework incorporating a bicyclo [2.2.2] octane moiety, which was proposed to arise through intramolecular Diles-Alder cyclization. Furthermore, single-crystal X-ray diffraction analysis pinpointed its absolute configuration. Biologically, compound **182** exhibited antiproliferative activity against LoVo colon carcinoma cells, with an IC_50_ value of 6.7 μM, whereas dimer **183** observed negligible cytotoxicity. Flow cytometry and Western blot analysis revealed that compound **182** induced apoptosis in LoVo cells [86].

Bislangduoids A and B (**184**,**185**) represent a novel class of dimeric *ent*-abietane diterpenoids isolated from the traditional Chinese medicinal plant *Euphorbia fischeriana* (Langdu). Both dimers were assembled from two distinct *ent*-abietane monomers through a carbon-carbon linkage between C17 and C15′. Notably, compound **184** features a highly oxidized and architecturally complex cage-like pentacyclic core. Biosynthetically, the dimeric skeleton of compounds **184**,**185** are proposed to arise predominantly via Michael addition and acetal-formation reactions. Biologically, compound **184** displayed significant cytotoxicity against HepG2 hepatocellular carcinoma cells with an IC_50_ value of 7.4 μM, and induced apoptosis in the HepG2 cells [87].

Two *ent*-abietane diterpenoid dimers, biseuphoids A and B (**186**,**187**), were isolated from *Euphorbia fischeriana*. These dimers exhibit rare structural connectivity, with biseuphoid A featuring a C-17 to C-12′ linkage and biseuphoid B featuring a C-17 to C-11′ connection, underscoring their distinctive dimerization patterns. Key Michael addition reaction is putatively responsible for the formation of these two dimers, providing valuable insights into their biosynthetic origins. Compounds **186**,**187** showed inhibitory activities against soluble epoxide hydrolase (sEH), with IC_50_ values of 8.17 μM and 5.61 μM, respectively. Additionally, the in silico molecular dynamics simulations revealed that both compounds **186** and **187** anchor within the catalytic pocket of sEH through stable hydrogen bond interaction with key amino acid residues, including Gln384, Asn378, Pro361, Ala365, Asn366, and Asn472, providing a structural rationale for their inhibitory potency [88].

### 2.5. Miscellaneous Ent-Abietane Diterpenoids

**Figure 16:**
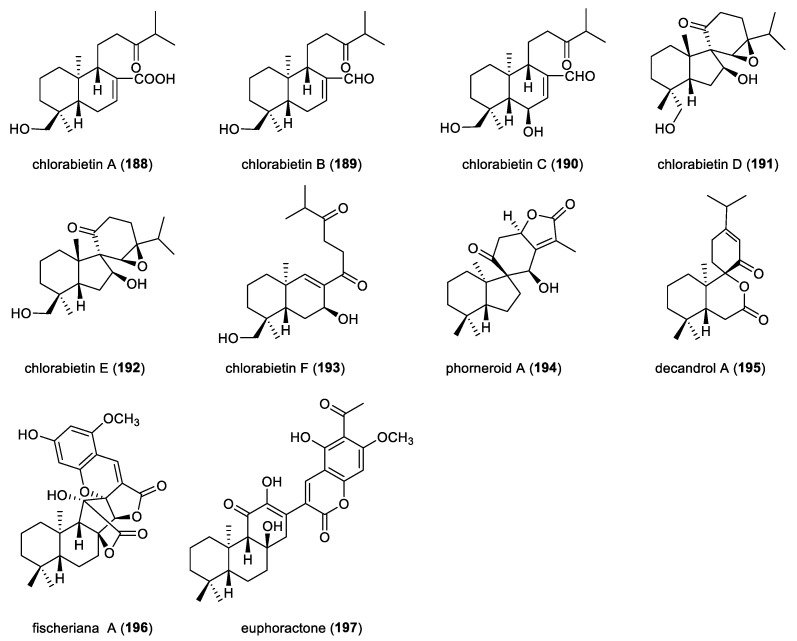
Chemical structures of miscellaneous *ent*-abietane diterpenoids (**188**–**197**).

Six *ent*-abietane diterpenoids, chlorabietins A–F (**188**–**193**), were isolated from the roots of *Chloranthus oldhamii* (Figure 16). The absolute configuration of compound **188** was established by X-ray crystallographic analysis. Compounds **188**–**190** feature rare 13,14-*seco*-*ent*-abietane derivatives, while compounds **191**,**192** represent the first example of 9,10-*seco*-8-spirofused-*ent*-abietane diterpenoids characterized by an unusual *cis*-fused A/B ring junction [22]. Compound **193** is a rare chinane-type skeleton resulting from C-ring cleavage between C-13 and C-14 and is more appropriately classified as a rearranged *ent*-abietane diterpenoid from a biosynthetic perspective. Compounds **189**,**190** and **193** exhibited anti-neuroinflammatory activities by inhibiting NO production in LPS-activated murine BV-2 microglial cells, with IC_50_ values ranging from 16.4 to 33.8 μM.

Phorneroid A (**194**), obtained from the aerial parts of *Euphorbia neriifolia*, represents the first example of 8-spirofused 9,10-seco-*ent*-abietane diterpenoid lactone featuring a unique 6/5/6/5 spirocyclic framework. The absolute configuration of compound **194** was determined by ECD calculations and single-crystal X-ray crystallographic analysis. Compounds **194** exhibited moderate cytotoxicity against HL-60 tumor cell lines, with an IC_50_ value of 9.9 μM [61]. Decandrol A (**195**), a rare C9-spirofused 7,8-*seco*-*ent*-abietane diterpenoid, was isolated from the roots of *Ceriops decandra*, an Indian mangrove collected from the swamp of Godavari estuary. The absolute configuration of the compound **195** was determined by ECD calculations [26]. Fischeriana A (**196**), isolated from the roots of *Euphorbia fischeriana*, represents a structurally distinctive meroterpenoid bearing a heptacyclic 6/6/5/5/5/6/6 scaffold arising from the fusion of a modified *ent*-abietane diterpene core with a phloroglucinol unit. Its absolute configuration was determined by single-crystal X-ray diffraction analysis. Compound **196** showed cytotoxicity against the HepG2 cells, with an IC_50_ value of 15.75 μM, comparable to the positive control cisplatin [89]. Euphoractone (**197**), a meroterpenoid integrating an *ent*-abietane moiety with phloroglucinol unit, was also isolated from the roots of *Euphorbia fischeriana*. The structural characterization of compound **197** was achieved by X-ray crystallography analysis. Compound **197** exhibited inhibitory activity against H23 and H460 human lung cancer cell lines, with IC_50_ values of 21.07 and 20.91 μM, respectively [90].

## 3. Synthesis of *Ent*-Abietane Diterpenoids

Owing to their remarkable structural diversity and significant biological activities, *ent*-abietane diterpenoids have been compelling synthetic targets. A seminal review has previously summarized total synthesis of these *ent*-abietane diterpenoids prior to 2015 [8]. Building on the previous review, this review is dedicated to highlighting notable advances in this field from 2016 to early 2025.

**Figure 17:**
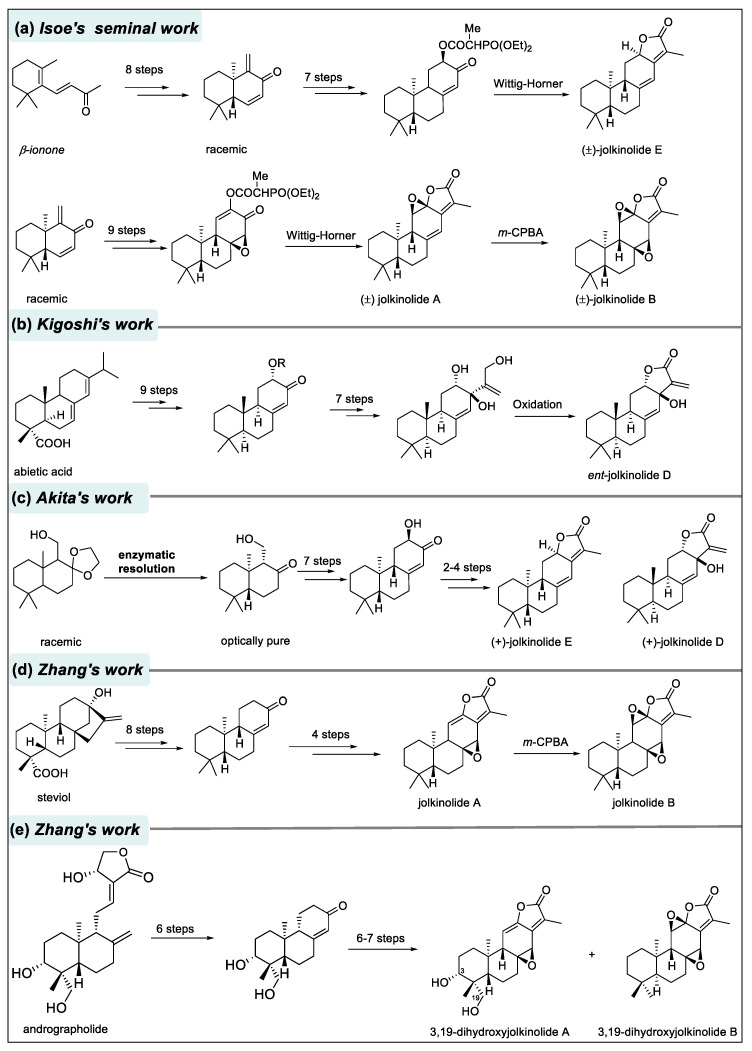
Overview of total synthesis of *ent*-abietane diterpenoids.

Three *ent*-abietane diterpenoid lactones, jolkinolides A, B and E, firstly isolated from the roots of *Euphorbia jolkini* in 1972, harbor a *γ*-butenolide motif [19]. Their intriguing structures promoted them attractive synthetic targets for organic chemistry, ultimately culminating in successful total synthesis of their racemic forms. Since the first total synthesis of jolkinolides A,B and E was achieved by Isoe and co-workers (Figure 17) [91], considerable research efforts have been directed toward the synthesis of *ent*-abietane diterpenoids. The following section outlines representative synthetic routes reported prior to 2015.

Kigoshi and co-workers utilized abietic acid as a chiral pool building block to access the enantiomer of jolkinolide D in 2004 [92]. It is noteworthy that the enantiomer lacked the biological activity observed for the natural jolkinolide D, mirroring the importance of stereochemistry in determining the bioactivity of diterpenoids. Subsequently, Akita and co-workers developed a chemoenzymatic route to the total synthesis of (+)-jolkinoides D and E in 2007 [93]. Zhang and co-workers accomplished total synthesis of jolkinolides A and B from readily available steviol in 2014 [94], and later synthesized jolkinolide derivatives, 3,19-dihydroxyjolkinolides using andrographolide as the starting material [95]. More recently, Tao’s team employed an intramolecular *oxa*-Pauson-Khand reaction (*o*-PKR) as the key step to effectively construct the 6/6/6/5 tetracyclic abietane diterpenoid skeleton, synthesizing the enantiomers of euphopilolide and jolkinolide E in just 11 and 12 steps, respectively [96].

**Figure 18:**
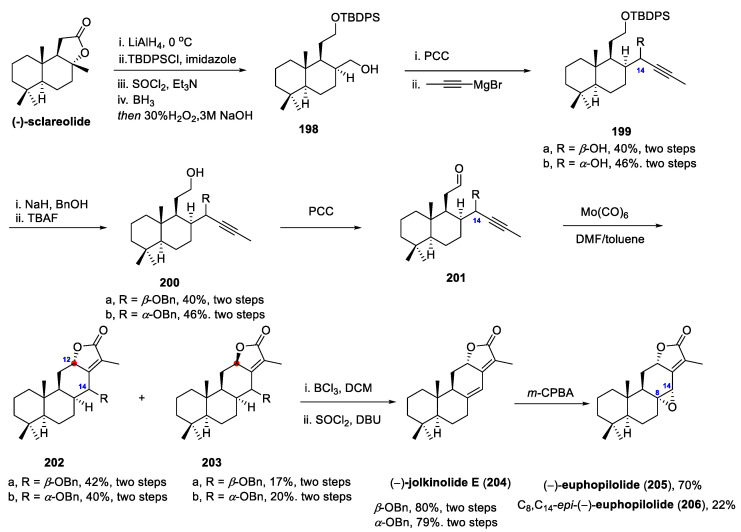
Tao’s total synthesis of (-)-jolkinolide E and (-)-euphopilolide.

Tao and co-workers initiated their synthesis from commercially available sclareolide (Figure 18), which in four steps arrives at the alcohol **198** via reduction in the lactone moiety with lithium aluminum hydride (LiAlH_4_), silyl ether protection of the resulting primary alcohol and a dehydration/hydroboration/oxidation sequence. Subsequent oxidation of this alcohol **198** with PCC yielded an aldehyde, which upon a Grignard addition with 1-propynylmagnesium bromide, provided a pair of C-14 alkyne epimers (**199**a,**199**b) in good overall yield over these two steps. Subsequent synthetic studies demonstrated that configuration of at C-14 was inconsequential to the overall yield, as a later *β*-hydride elimination step equilibrated the epimers *en route* to the final target molecule, jolkinolide E.

Alkyne **199**a and **199**b were then elaborated to the *o*-PKR precursor **201** via a three-step sequence comprising benzyl protection of the secondary alcohol, removal of the TBDPS protecting group, and PCC-mediated oxidation. In practice, the key cyclization precursor **201** was found to be unstable and readily underwent an intramolecular *o*-PKR under stoichiometric Mo(CO)_6_ in a refluxing binary solvent system (DMF and toluene). This transformation furnished the desired *γ*-butenolide-fused tetracyclic core characteristic of *ent*-abietane diterpenoids as a pair of C12 epimers in 59% combined yield [97]. The major product (**202**a and **202**b) possessed the required configuration at C12, consistent with that of the natural products. In parallel, tetracyclic butenolides (**203**a, **203**b) were both prepared via a conceptually analogous route, thus unveiling that epimerization at C-14 had negligible impact on the efficiency of *o*-PKR step in this setting. Subsequent treatment of compound **202**a and **202**b with a Lewis acid promoted benzyl deprotection, liberating a secondary alcohol that underwent *β*-hydride elimination to afford the enantiomer of natural product jolkinolide E (**204**). Moreover, jolkinolide E was transformed into the enantiomer of euphopilolide (**205**) via epoxidation, yielding a separable minor diastereomer identified as C8, C14-*epi*-(-)-euphopilolide (**206**).

## 4. Biological Activity

*Ent*-abietane diterpenoids form a highly diverse family of natural terpenoids widely distributed across various plant genera, including *Euphorbia*, *Chloranthus*, *Isodon*, *Ceriops*, *Croton*, *Suregada* and *Phlogacanthus*. Owing to their rich structural variability and promising bioactivity profiles, these compounds have garnered considerable attention in natural product chemistry. They exhibit a wide array of biological properties, most notably, anticancer, anti-inflammatory, antibacterial, and neuroprotective activities, providing a new window for the future drug discovery [98,99,100,101].

### 4.1. Anticancer Activity

Anticancer activity represents one of the most prominent biological properties of *ent*-abietane diterpenoids, with many members exhibiting potent cytotoxic, pro-apoptotic or cancer stem cell targeting effects across a wide spectrum of tumor types. Notably, several compounds also exhibit remarkable activity against drug-resistant cancer phenotypes, underscoring their therapeutic potential.

A number of *ent*-abietane diterpenoids exhibit broad-spectrum cytotoxicity. Phyllostachysins K and L, for example, display moderate cytotoxicity against several cell lines including HL-60 (leukemia), SMMC-7721 (hepatocellular carcinoma), A-549 (lung cancer), MCF-7 (breast cancer), and SW-480 (colorectal cancer), with IC_50_ values ranging from 4.1 to 29.8 μM [23]. Similarly, isoforrethins C and D display IC_50_ values of 10.1–20.2 μM against SW-80 (colorectal cancer), HL-60, MCF-7, and A-549 cell lines. Among dimeric members, bisebracteolasins A and B show IC_50_ values in the range of 2.61–14.09 μM against a panel of cancer cell lines (HL-60, A549, SMMC-7721, MCF, and SW-480), demonstrating extensive tumor cell inhibitory activity [28].

Several *ent*-abietane diterpenoids demonstrate promising activity against prostate cancer cells, including drug-resistant phenotype. Euphonoid H exhibits significant antiproliferative activity against both the enzalutamide-sensitive prostate cancer cell line C4-2B and the enzalutamide-resistant cell line C4-2B/ENZR, with IC_50_ values of 5.52 μM and 4.16 μM, respectively [38]. Euphonoid I exhibits a comparable potency, with IC_50_ values of 4.49 and 5.74 μM against the aforementioned two cell lines. Euphonoids A and B also show inhibitory effects on these cell lines, with IC_50_ values of 9.18/9.7 μM and 13.4/11.1 μM, respectively. Euphopane B shows an IC_50_ value of 16.9 μM against the C4-2B prostate cancer cell line [30].

Several compounds show activity against breast cancer cells. Fischdiabietane A exhibits cytotoxicity against the T47D, with an IC_50_ value of 6.51 μM, displaying nearly sixfold higher than that of the positive control cisplatin. Mechanistic studies revealed that its activity involves apoptosis mediated by caspase-3 activation and PARP degradation [84]. In addition, euphorins E and H suppress mammosphere formation in MCF-7 breast cancer cells at 10 μM, suggesting a capacity to target breast cancer stem cells-like populations [21].

Hepatocellular carcinoma (HCC) is another target of *ent*-abietane diterpenoids. Bislangduoid A demonstrates cytotoxicity against the HepG2 hepatocellular carcinoma cell lines, with an IC_50_ value of 7.4 μM and induce cell apoptosis. Likewise, 3*α*-acetoxy-14-hydroxy-*ent*-abieta-8(9),13(15)-dien-16,12-olide exhibits moderate cytotoxicity against the SMMC-7721 cell line, with an IC_50_ value of 29.0 μM [87].

Beyond these categories, several *ent*-abietane diterpenoids have been reported to show cytotoxicity against additional cancer cells. Difischenoids A–B and euphorfinoids N, exhibit inhibitory effects against the HeLa cervical cancer cell line, with IC_50_ values of 15.47, 3.75 and 3.62 μM, respectively [35]. Biseupyiheoid A demonstrates cytotoxicity against LoVo colorectal cancer cell, with IC_50_ value of 6.7 μM [86]. Bisebracteolasins A and B exhibit inhibitory activity against the CD44^+^ colorectal cancer stem cell line P6C, with IC_50_ values of 16.48 and 34.76 μM, respectively [83]. Among them, bisebracteolasin A can further inhibit the tumorsphere formation and migration ability of P6C cells, suggesting therapeutic potential in suppressing cancer stem cell-mediated tumor growth and metastasis.

### 4.2. Anti-Inflammatory Activity

The anti-inflammatory properties of *ent*-abietane diterpenoids are primarily attributed to their ability to suppress the production of key inflammatory mediators and modulate central inflammatory signaling pathways, including nitric oxide, the nuclear factor-κB (NF-κB) and related cytokines, IL-6, IL-1*β*, and TNF-*α*.

A number of *ent*-abietane diterpenoids exhibit significant nitric oxide production in LPS-stimulated macrophage or microglial cell models. Among the most potent examples, ebractenoids L and N exhibit effects in RAW264.7 macrophages, with IC_50_ values of 0.69 and 1.97 μM, respectively [48]. Serrin K, phyllostachysins K and L also display strong inhibitory activity, with IC_50_ values of 1.8 μM, 1.34 μM, and 2.09 μM, respectively [24]. Additional contributors, euphohelinode A and euphohelide H show moderate inhibitory activity against RAW264.7 cells, with IC_50_ values of 32.98 and 30.23 μM, respectively [67].

Several diterpenoids show anti-inflammatory effects through inhibition of nitric oxide production and follow-up mechanistic analysis have shed light on the signal pathway responsible for these actions. Euphohelinode H is attributed to the inhibition of the NF-κB signaling pathway and the downregulation of pro-inflammatory enzymes COX-2 and iNOS [67]. Euphjatrophane M demonstrates a multifaceted regulatory profile, dose-dependently inhibiting the mRNA expression of IL-6, IL-1*β*, and TNF-*α* while concurrently reducing the expression of FOXO1 and the phosphorylation level of NF-κB p65 [68]. Decandrols C and E attenuate NF-κB activity at a concentration of 100 μM; the anti-inflammatory effects of euphohelide A are also speculated to involve NF-κB signaling pathway [26].

A subset of *ent*-abietane diterpenoids demonstrates notable activity in neuroinflammatory models. Chlorabietins B, C, F and G inhibit nitric oxide production in LPS-activated BV-2 microglial cells, with IC_50_ values ranging from 16.4 to 33.8 μM [22].

### 4.3. Antibacterial Activity

*Ent*-abietane diterpenoids displaying antibacterial activity have been reported against *Mycobacterium tuberculosis*, Gram-positive bacteria, and Gram-negative bacteria, with most compounds exhibiting moderate inhibitory potency.

Eupholides F–H exhibit moderate inhibitory activity against *Mycobacterium tuberculosis* H37Ra, with a MIC of 50 μM [31]. Several *ent*-abietane diterpenoids demonstrate activity against Gram-positive bacteria. Three compounds, 6*β*-hydroxy-*ent*-abieta-7,13-dien-3-one, 2*β*,13*α*,15-trihydroxy-*ent*-abieta-8(14)-en-3-one and 2*β*,9*α*,13*β*,15-tetrahydroxy-*ent*-abieta-7-en-3-one exhibit weak antibacterial activity against Gram-positive strains T25-17, C159-6, and sp.8152, with MIC values less than 50 μg/mL [36]. Similarly, 11*β*-hydroxy-14-oxo-17-al-*ent*-abieta-8(9),13(15)dien-16,12*β*-olide, 11*β*,17-dihydroxy-12-methoxy-*ent*-abieta-8(14),13(15)-dien-16,12*α*-olide, and 14*α*-hydroxy-17-al-*ent*-abieta-7(8),11(12),13(15)-trien-16,12-olide display MIC values under 60 μg/mL against the same bacterial strains [58]. Sureproceriolide A demonstrate moderate antibacterial activity against the Gram-positive bacterium *Staphylococcus lugdunensis*, with an MIC value of 31.44 μM [63].

### 4.4. Other Biological Activities

Beyond cytotoxic, anti-inflammatory and antibacterial effects, several *ent*-abietane diterpenoids show activity profiles that broaden their therapeutic potential. Abientaphlogatone D and E exhibits neuroprotective activity in PC12 cell injury models induced by H_2_O_2_ and MPP^+^, and SAR analysis highlights the important role of the hydroxyl group in the aromatic C-ring [47]. Dimeric *ent*-abietane diterpenoids, biseuphoids A and B serve as inhibitors of soluble epoxide hydrolase (sEH), with IC_50_ values ranging from 5.61 to 10.29 μM. In silico studies reveals that these compounds anchor within the enzyme’s catalytic pocket, forming stable hydrogen bonds with key amino acid residues such as Gln384 and Asn378. This class of compounds holds promise for the treatment of inflammation-related diseases [85]. Euphorfinoid L exhibits weak inhibitory activity against acetylcholinesterase (AChE) with an IC_50_ value of 147.51 μM [70].

## 5. Conclusions

Over the past decade, substantial progress has been made in the isolation and structural characterization of *ent*-abietane diterpenoids. Key advances include the expansion of plant sources from which these compounds have been obtained. Prior to 2015, most *ent*-abietane diterpenoids were identified from the genus *Euphorbia* (Euphorbiaceae). In recent years, however, *ent*-abietane diterpenoids have also been discovered from species belonging to the *Chloranthaceae*, *Rhizophoraceae*, *Labiatae* and *Asteraceae* families, thereby enriching the diversity of plant resources and laying a foundation for the isolation of more structurally diverse members of this class.

Furthermore, the discovery of norditerpenoids, rearranged diterpenoids and dimeric diterpenoids greatly diversifies the chemical space of this family, offering new opportunities for drug discovery. Although recent total synthesis of two *ent*-abietane diterpenoids have been achieved, the synthesis predominately focused on a subclass. More challenging targets, such as dimeric or rearranged diterpenoids, have yet to be conquered. Moreover, certain members of the *ent*-abietane diterpenoid family have exhibited promising inhibitory activity against soluble epoxide hydrolase (sEH), underscoring their potential as a valuable source for therapeutic leads.

## 6. Future Perspectives

The future of *ent*-abietane diterpenoid research hinges on adopting a multifaceted and integrative approach. Continued exploration of more structurally diverse natural products within this family holds great promise of advancing natural products-based drug discovery. Strengthening the chemical synthesis of these diterpenoids will help overcome the limited availability of materials isolated from natural sources. New synthetic strategies should allow for catalytic asymmetric total synthesis of these diterpenoids, facilitating the preparation of sufficient quantities of compounds for comprehensive biological evaluation. Despite the absence of AI-assisted synthetic studies specifically targeting *ent*-abietane diterpenoids, the rapid progress in data-driven organic synthesis suggests that closely related investigations may be reported in the foreseeable future.

At present, research on *ent*-abietane diterpenoids is routinely located at fragmented or uncoordinated efforts. Specifically, although isolation of new *ent*-abietane diterpenoids is frequently accompanied by preliminary biological screening, systematic scaffold simplification and functional group modification aimed at improving biological profiles remain largely underexplored. Therefore, it is necessary to shift from dispersed individual studies toward coordinated, well-supported and interdisciplinary programs in which isolation, chemical synthesis and in-depth biological investigation are seamlessly integrated. Such a unified strategy will not only broaden our understanding of this chemically rich family but also establish a new paradigm for natural product discovery and development.

## Data Availability

All the data and materials provided in this manuscript were obtained from references and available upon request.

## Supplementary Materials

The following supporting information can be downloaded at: https://www.mdpi.com/article/10.3390/molecules31010098/s1, Table S1 Compound names, plant sources and their reported activities of prototype *ent*-abietane diterpenoids; Table S2 Compound names, plant sources and their reported activities of aromatic *ent*-abietane diterpenoids; Table S3 Compound names, plant sources and their reported activities of *ent*-abietane diterpenoid lactones; Table S4 Compound names, plant sources and their reported activities of dimeric *ent*-abietane diterpenoids; Table S5 Compound names, plant sources and their reported activities of miscellaneous *ent*-abietane diterpenoids.

## Abbreviations

ECD	electronic circular dichroism
IC_50_	half maximal inhibitory concentration
EC_50_	half maximal effective concentration
LPS	lipopolysaccharide
MIC	minimum inhibitory concentration
SAR	Structure–activity relationship
