# Supplementary material for: Recent Advances in Ent-Abietane Diterpenes: Natural Sources, Biological Activities and Total Synthesis
Source: Molecules. 2025 Dec 25;31(1):98. doi: 10.3390/molecules31010098 (PMC12786761; doi:10.3390/molecules31010098)
Supplement: Supplementary file 1 [file molecules-31-00098-s001.zip › molecules-4047017-supplementary.pdf]

Supplementary File

Recent Advances in *ent*-Abietane Diterpenes: Natural Sources, Biological Activities and Total Synthesis

Lu Li[1†] , Yongjie Zhu[1†], Haixia Deng[1], Liqiong Xie[1], Chang-Bo Zheng[1,2], Jian-Neng Yao[1,2*] and Ji Li[1,2*]

[1]*School of Pharmaceutical Sciences and Yunnan Key Laboratory of Pharmacology for Natural Products, Kunming Medical University, Kunming, Yunnan 650500, China;*

[2]*Yunnan College of Modern Biomedical Industry, Kunming Medical University, Kunming, Yunnan 650500, China;* .

* Correspondence authors, Email: gigiliji@163.com (Ji Li);

yaojianneng@kmmu.edu.cn (Jian-Neng Yao).

†These authors contribute equally to this work.

Index

Table S1 Compound names, plant sources and their reported activities of prototype *ent*-abietane diterpenoids **S3**

Table S2 Compound names, plant sources and their reported activities of aromatic *ent*-abietane diterpenoids **S6**

Table S3 Compound names, plant sources and their reported activities of *ent*-abietane diterpenoid lactones **S8**

Table S4 Compound names, plant sources and their reported activities of dimeric *ent*-abietane diterpenoids **S16**

Table S5 Compound names, plant sources and their reported activities of miscellaneous *ent*-abietane diterpenoids **S17**

References **S17**

Table S1 Compound names, plant sources and their reported activities of prototype *ent*-abietane diterpenoids

Compound name	Plant sources	Cell lines/Test model	IC_{50}	Reported activities	Reference*
euphorin H **(1)**	*Euphorbia fischeriana*	MCF-7	10 μM	cytotoxicity activity	21
chlorabietin G **(2)**	*Chloranthus oldhamiia*	LPS-activated murine BV-2 microglial cells	23.8 μM	anti-inflammatory activity	22
chlorabietin H **(3)**	*Chloranthus oldhamiia*				22
chlorabietin I **(4)**	*Chloranthus oldhamiia*				22
phyllostachysin K **(5)**	*Isodon phyllostachys*	human tumor cell lines (HL-60 SMMC-7721, A-549, MCF-7,SW-480)	4.1-29.8 μM	cytotoxicity activity	23
		RAW264.7 cells stimulated by LPS	1.34 μM	anti-inflammatory activity	
phyllostachysin L **(6)**	*Isodon phyllostachys*	human tumor cell lines (HL-60 SMMC-7721, A-549, MCF-7, SW-480)	4.1-29.8 μM;	cytotoxicity activity	23
		RAW264.7 cells stimulated by LPS	2.09 μM	anti-inflammatory activity	
serrin K **(7)**	*Isodon serra*	RAW264.7 cells stimulated by LPS	1.8 μM	anti-inflammatory activity	24
xerophilusin XVII **(8)**	*Isodon serra*				24
enanderianins Q **(9)**	*Isodon serra*				24
enanderianins R **(10)**	*Isodon serra*				24
fischeriabietane A **(11)**	*Euphorbia fischeriana*				25
fischeriabietane B **(12)**	*Euphorbia fischeriana*	Panc-28	47.2 μM	cytotoxicity activity	25
fischeriabietane C **(13)**	*Euphorbia fischeriana*	Bel-7402	12.9 μM	cytotoxicity activity	25
		Panc-28	20.7 μM		
decandrol G **(14)**	*Ceriops decandra*				26
decandrol H **(15)**	*Ceriops decandra*				26

decandrol I **(16)**	*Ceriops decandra*				26
raserrane A **(17)**	*Rabdosia serra*				27
raserrane B **(18)**	*Rabdosia serra*				27
isoforrethin A **(19)**	*Isodon forrestii*				28
isoforrethin B **(20)**	*Isodon forrestii*				28
isoforrethin C **(21)**	*Isodon forrestii*	SW-80,HL-60,MCF-7,A-549	10.1- 20.2 μM	cytotoxicity activity	28
isoforrethin D **(22)**	*Isodon forrestii*	SW-80, HL-60, MCF-7, A-549	10.1- 20.2 μM	cytotoxicity activity	28
(2*R*,3*S*,5*S*,9*S*,10*R*)-2,3-dihydroxy-*ent*-abieta-8(14),12(13)-dien-7 -one**(23)**	*Euphorbia thymifolia*				29
euphonoid C **(24)**	*Euphorbia fischeriana*				30
euphonoid D **(25)**	*Euphorbia fischeriana*				30
eupholide H **(26)**	*E. fischeriana*	*Mycobacterium tuberculosis* H37Ra	50 μM (MIC)	anti-mycobacterial activity	31
2*β*-hydroxy-*ent*-abieta-7,13-dien-3-one **(27)**	*Croton mubango*				32
15-hydroxy-*ent*-abieta-7, 13-dien3-one **(28)**	*Croton mubango*				32
13*α*,15-dihydroxy-*ent*-abieta-8(14)-en-3-one **(29)**	*Croton mubango*				32
2*β*,9,13-trihydroxy-*ent*-abieta-7-en-3-one **(30)**	*Croton mubango*				32

euphopane B **(31)**	*Euphorbia pekinensis*	C4-2B	16.9 μM	cytotoxicity activity	34
difischenoid A **(32)**	*Euphorbia fischeriana*	Hela	15.47 μM	cytotoxicity activity	35
6β-hydroxy-*ent*-abieta-7,13-dien-3-one **(33)**	*Croton cascarilloide*	Gram positive bacteria T25-17, C159-6,sp.8152	<50 μg /ml	antibacterial activity	36
2β,13α,15-trihydroxy-*ent*-abieta-8(14)-en-3-one **(34)**	*Croton cascarilloide*	Gram positive bacteria T25-17, C159-6,sp.8152	<50 μg /ml	antibacterial activity	36
2β,9α,13β,15-tetrahydroxy-*ent*-abieta-7-en-3-one **(35)**	*Croton cascarilloide*	Gram positive bacteria T25-17, C159-6,sp.8152	<50 μg /ml	antibacterial activity	36
7β,13α,15-trihydroxy-*ent*-abieta-8(14)-en-3-one **(36)**	*Croton lachnocarpus*				37
euphonoid H **(37)**	*Euphorbia fischeriana*	C4-2B	5.52 μM	cytotoxicity activity	38
		C4-2B/ENZR	4.16 μM		
isogeopyxin C **(38)**	*Isodon parvifolia*				39
(7*R*,8*S*)-7,8-dihydroxy-17-nor-*ent*-abieta13(14)-en-15-one **(39)**	*Euphorbia jolkinii*				40
isodopene A **(40)**	*Isodon ternifolius*	DNA topoisomerase IB (TOP1)		TOP1 inhibitory activity	41
henanabinin A **(41)**	*Isodon rubescens*				42
henanabinin B **(42)**	*Isodon rubescens*				42
henanabinin C **(43)**	*Isodon rubescens*				42
lathyrisol B **(44)**	*Euphorbia lathyrism*				43

*All references listed in the Supplementary File are included in the main text and the corresponding reference numbers in the last column are consistent with those in

the main text.

Table S2 Compound names, plant sources and their reported activities of aromatic *ent*-abietane diterpenoids

Compound name	Plant sources	Cell lines/Test model	IC_{50}	Reported activities	Reference*
chlorabietin J **(45)**	*Chloranthus oldhamii.*				22
chlorabietin K **(46)**	*Chloranthus oldhamii.*				22
chlorabietin L **(47)**	*Ceriops decandra*				22
decandrol B **(48)**	*Ceriops decandra*				26
decandrol C **(49)**	*Ceriops decandra*	RAW264.7 cells stimulated by LPS (NF-κB)	100 μM	anti-inflammatory activity	26
decandrol D **(50)**	*Ceriops decandra*				26
decandrol E **(51)**	*Ceriops decandra*	RAW264.7 cells stimulated by LPS (NF-κB)	100 μM	anti-inflammatory activity	26
decandrol F **(52)**	*Ceriops decandra*				26
ent-abieta-8,11,13-trien-3-one **(53)**	*Croton mubango*				37
7*β*-hydroxy-*ent*-abieta-8,11,13-trien-3-one **(54)**	*Croton mubango*				37
2*β*,7*β*-dihydroxy-*ent*-abieta-8,11,13-trien-3-one **(55)**	*Croton mubango*				37
15-hydroxy-*ent*-abieta-8,11,13-trien-3-one **(56)**	*Croton mubango*				37

3*α*-hydroxy-*ent*-abieta-8,11,13-triene **(57)**	*Croton mubango*				37
15-hydroxy-*ent*-abieta-8,11,13triene **(58)**	*Croton mubango*				37
6*β*-hydroxy-*ent*-abieta-8,11,13-triene **(59)**	*Croton mubango*				37
7*β*,15-dihydroxy-*ent*-abieta-8,11,13-trien-3-one **(60)**	*Croton lachnocarpus*				37
2*β*,15-dihydroxy-*ent*-abieta-8,11,13-triene **(61)d**	*Croton lachnocarpus*				37
leucoabietene A **(62)**	*Candida albicans*	fluconazole-resistant *Candida albicans*	32 µg/mL (threshold concentrat ion)	chemical sensitizing activity	44
euphomauritanol A **(63)**	*Euphorbia mauritanica*	B16-BL6	10.28 µM	antiproliferative activity	45
euphomauritanol B **(64)**	*Euphorbia mauritanica*	B16-BL6	20.22 µM	antiproliferative activity	45
forsyditerpene N **(65)**	*Forsythia suspensa*				46
forsyditerpene O **(66)**	*Forsythia suspensa*				46
Wallichane H **(67)**	*Euphorbia wallichii*				46
abientaphlogatones E **(68)**	*Phlogacanthus curviflorus*				47
abientaphlogatones F **(69)**	*Phlogacanthus curviflorus*	PC12 injury by H_2O_2 and MPP^+		neuroprotective activity	47

*All references listed in the Supplementary File are included in the main text and the corresponding reference numbers in the last column are consistent with those in the main text.

Table S3 Compound names, plant sources and their reported activities of *ent*-abietane diterpenoid lactones

Compound name	Plant sources	Cell lines/Test model	IC_{50}	Reported activities	Reference*
ebractenoid K **(70)**<br>euphrin F	*Euphorbia ebracteolata*				48
ebractenoid L **(71)**<br>euphrin G	*Euphorbia ebracteolata*	RAW264.7 cells stimulated by LPS	0.69 μM	anti-inflammatory activity	48
ebractenoid M **(72)**	*Euphorbia ebracteolata*				48
ebractenoid N **(73)**	*Euphorbia ebracteolata*	RAW264.7 cells stimulated by LPS	1.97 μM	anti-inflammatory activity	48
euphorin E **(74)**	*Euphorbia fischeriana*	MCF-7	10 μM	cytotoxicity activity	49
11*α*,17-dihydroxyhelioscopinolide E **(75)**	*Euphorbia fischeriana*				49
6*β*,11*α*,17-trihydroxyhelioscopinolide E **(76)**	*Euphorbia fischeriana*				49
11-*oxo*-ebracteolatanolide B **(77)**	*Euphorbia fischeriana*				49
7-deoxylangduin B **(78)**	*Euphorbia fischeriana*				49
fischeriabietane D **(79)**	*Euphorbia fischeriana*				25
fischeriabietane E **(80)**	*Euphorbia fischeriana*				25
euphoroid A **(81)**	*Euphorbia ebracteolate*				50
euphoroid B **(82)**	*Euphorbia ebracteolate*				50
euphoroid C **(83)**	*Euphorbia ebracteolate*	A549,MCF-7,Lovo,SH-SY5Y	<30 μM	cytotoxicity activity	50
ebracteolata D **(84)**	*Euphorbia ebracteolata*				51
mangiolide **(85)**	*Suregada zanzibariensis*	TK10	(TGI)	antitumor activity	52

			0.07 µg/ml (GI$_{50}$) 0.02 µg/ml		
		UACC62	(TGI) 0.06 µg/ml (GI$_{50}$) 0.03 µg/ml		
		MCF7	(TGI) 0.33 µg/ml (GI$_{50}$) 0.05 µg/ml		
eupneria A **(86)**	*Euphorbia neriifolia*				53
eupneria B **(87)**	*Euphorbia neriifolia*				53
eupneria C **(88)**	*Euphorbia neriifolia*				53
eupneria D **(89)**	*Euphorbia neriifolia*				53
eupneria E **(90)**	*Euphorbia neriifolia*				53
eupneria F **(91)**	*Euphorbia neriifolia*				53
(1*S*,5*R*,9*R*,10*R*,12*R*)-1*α*-acetoyloxy-*ent*-abieta-8(14),13(15)-dien-12a,16-olide **(92)**	*Euphorbia royleana*	LPS-activated murine BV-2 microglial cells	12.0 µM	anti-inflammatory activity	54
(1*S*,4*S*,5*R*,9*R*,10*S*,12*R*)-18*β*-methylenedioxy-*ent*-abieta-8(14),13(15)-dien-12*α*,16-olide （**93**）	*Euphorbia royleana*				54
euphonoid A **(94)**	*Euphorbia fischeriana*	C4-2B	9.18 µM	cytotoxicity activity	30

		C4-2B/ENZR	9.7 μM		
euphonoid B **(95)**	*Euphorbia fischeriana*	C4-2B	13.4μM	cytotoxicity activity	30
		C4-2B/ENZR	11.1 μM		
euphcopenoid A **(96)**	*Euphorbia helioscopia*				55
euphcopenoid B **(97)**	*Euphorbia helioscopia*				55
11,12-didehydro-8*α*,14-dihydro-7-*oxo*-helioscopinolide A **(98)**	*Euphorbia peplus*				56
7*α*-hydroxy-8*α*,14-dihydro jolkinolide E **(99)**	*Euphorbia peplus*				56
7*α*-hydroxy-*ent*-abieta-8(14),13(15)-dien-16,12*β*-olide **(100)**	*Baccharis sphenophylla*	NCTC 929	<200 μM(CC_{50}) >9.4(SI)	high bioactivity and low cytotoxicity	57
11*β*-hydroxy-14-oxo-17-al-*ent*-abieta-8(9),13(15)dien-16,12*β*-olide **(101)**	*Euphorbia wallichii*	*Gram-positive bacteria* (T25-17,C159-6,sp.8152)	<60 μg/mL(MIC)	antibacterial activity	58
11*β*,17-dihydroxy-12-methoxy-*ent*-abieta-8(14),13(15)-dien-16,12*α*-olide **(102)**	*Euphorbia wallichii*	*Gram-positive bacteria* (T25-17,C159-6,sp.8152)	<60 μg/mL(MIC)	antibacterial activity	58
14*α*-hydroxy-17-al-*ent*-abieta-7(8),11(12),13(15)-trien-16,12-olide **(103)**	*Euphorbia wallichii*	*Gram-positive bacteria* (T25-17,C159-6,sp.8152)	<60 μg/mL(MIC)	antibacterial activity	58
euphonoid F **(104)**	*Euphorbia antiquorum*				59
3-oxojolkinolide A **(105)**	*Glycosmis pentaphylla*				60
phorneroid B **(106)**	*Euphorbia neriifolia*	A549,HL-60	2.5-9.0 μM	cytotoxicity activity	61
phorneroid C **(107)**	*Euphorbia neriifolia*	A549,HL-60	2.5-9.0 μM	cytotoxicity activity	61
phorneroid D **(108)**	*Euphorbia neriifolia*				61

euphejolkinolide A **(109)**	*Euphorbia peplus*	lysosome biogenesis and autophagy mediated by EB		antitumor activity	62
sureproceriolide A **(110)**	*Suregada procera*	SK-MEL-28	23.50 μM	tumor-selective cytotoxic activity	63
		CCD-13Lu	27.90 μM		
		VCaP	35.14 μM		
		Staphylococcus lugdunensis	31.44 μM(MIC)	antibacterial activity	
		RAW264.7 cells stimulated by LPS (TNF-α)	3.31-5.16 μM	anti-inflammatory activity	
		RAW264.7 cells stimulated by LPS (NF-κB)	13.68-22.80 μM		
euphonoid I **(111)**	*Euphorbia fischeriana*	C4-2B	4.49μM	cytotoxicity activity	38
		C4-2B/ENZR	5.74 μM		
abientaphlogatone A **(112)**	*Phlogacanthus curviflorus*				47
abientaphlogatone B **(113)**	*Phlogacanthus curviflorus*	*β*-hematin formation inhibition	22.85 μM	antimalarial activity	47
abientaphlogatone C **(114)**	*Phlogacanthus curviflorus*				47
abientaphlogatone D **(115)**	*Phlogacanthus curviflorus*	*β*-hematin formation inhibition	14.21 μM	antimalarial activity	47
		PC12 injury by H_2O_2 and MPP^+	20 and 50 $\mu mol \cdot L^{-1}$	neuroprotective activity	
11*β*,12*β*-dihydroxy-*ent*-abieta-8(14),13(15)-dien-16,12*α*-olide **(116)**	*Euphorbia jolkinii*				64
(1*S*,3*S*)-1,3-dihydroxy-*ent*-abieta-	*Euphorbia jolkinii*				64

8(14),13(15)dien-17,12-olide **(117)**					
3*α*-acetoxy-14-hydroxy-*ent*-abieta-8(9),13(15)-dien-16,12-olide **(118)**	*Euphorbia fischeriana*	HL-60	15.3 μM	cytotoxicity activity	64
		SMMC-7721	29.0 μM		
3*α*,7*β*-dihydroxy-*ent*-abieta-11(12),13(15)-dien-16,12-olide **(119)**	*Euphorbia fischeriana*				64
2*β*-hydroxy helioscopinolide B **(120)**	*Euphorbia fischeriana*				64
banyangmbolide A **(121)**	*Suregada occidentalis*				65
banyangmbolide B **(122)**	*Suregada occidentalis*				65
banyangmbolide C **(123)**	*Suregada occidentalis*				65
banyangmbolide D **(124)**	*Suregada occidentalis*				65
banyangmbolide E **(125)**	*Suregada occidentalis*				65
spinidensolide A **(126)**	*Euphorbia spinidens*				66
euphohelinode D **(127)**	*Euphorbia helioscopia*				67
euphohelinode E **(128)**	*Euphorbia helioscopia*				67
euphohelinode F **(129)**	*Euphorbia helioscopia*				67
euphohelinode G **(130)**	*Euphorbia helioscopia*				67
euphohelinode H **(131)**	*Euphorbia helioscopia*	RAW264.7 cells stimulated by LPS (NF-κB, COX-2,iNOS)	30.23 μM	anti-inflammatory activity	67
euphohelinode I **(132)**	*Euphorbia helioscopia*				67
euphjatrophane H **(133)**	*Euphorbia peplus*				68
euphjatrophane I **(134)**	*Euphorbia peplus*				68

euphjatrophane J **(135)**	*Euphorbia peplus*				68
euphjatrophane K **(136)**	*Euphorbia peplus*				68
euphjatrophane L **(137)**	*Euphorbia peplus*				68
euphjatrophane M **(138)**	*Euphorbia peplus*	RAW264.7 cells stimulated by LPS (IL-6, IL-1β, and TNF-α, FOXO1,NF-κB p65)	10 μM	anti-inflammatory activity	68
euphjatrophane N **(139)**	*Euphorbia peplus*				68
euphjatrophane O **(140)**	*Euphorbia peplus*				68
euphjatrophane P **(141)**	*Euphorbia peplus*	RAW264.7 cells stimulated by LPS (IL-6, IL-1β, and TNF-α, FOXO1,NF-κB p65)	10 μM	anti-inflammatory activity	68
euphjatrophane Q **(142)**	*Euphorbia peplus*				68
euphjatrophane R **(143)**	*Euphorbia peplus*	RAW264.7 cells stimulated by LPS (IL-6, IL-1β, and TNF-α, FOXO1,NF-κB p65)	10 μM	anti-inflammatory activity	68
eupholide A **(144)**	*Euphorbia fischeriana*				31
eupholide B **(145)**	*Euphorbia fischeriana*				31
eupholide C **(146)**	*Euphorbia fischeriana*				31
eupholide D **(147)**	*Euphorbia fischeriana*				31
eupholide E **(148)**	*Euphorbia fischeriana*				31
eupholide F **(149)**	*Euphorbia fischeriana*	*Mycobacterium tuberculosis* H37Ra	50 μM (MIC)	anti-mycobacterial activity	31
eupholide G **(150)**	*Euphorbia fischeriana*	*Mycobacterium tuberculosis* H37Ra	50 μM (MIC)	anti-mycobacterial activity	31
		HCE 2	7.3 nM	metabolic regulatory potential	
euphelionolide A **(151)**	*Euphorbia helioscopia*				69

euphelionolide B **(152)**	*Euphorbia helioscopia*				69
euphelionolide C **(153)**	*Euphorbia helioscopia*				69
euphelionolide D **(154)**	*Euphorbia helioscopia*				69
euphelionolide E **(155)**	*Euphorbia helioscopia*				69
euphelionolide F **(156)**	*Euphorbia helioscopia*	MCF-7,PANC-1	9.5-10.7 μM	cytotoxicity activity	69
euphelionolide G **(157)**	*Euphorbia helioscopia*				69
euphelionolide H **(158)**	*Euphorbia helioscopia*				69
euphelionolide I **(159)**	*Euphorbia helioscopia*				69
euphelionolide J **(160)**	*Euphorbia helioscopia*				69
euphelionolide K **(161)**	*Euphorbia helioscopia*				69
euphelionolide L **(162)**	*Euphorbia helioscopia*				69
euphelionolide M **(163)**	*Euphorbia helioscopia*				69
euphelionolide N **(164)**	*Euphorbia helioscopia*	MCF-7,PANC-1	9.5-10.7 μM	cytotoxicity activity	69
euphorfinoid L **(165)**	*Euphorbia fischeriana*	AChE	147.51 μM	acetylcholinesterase inhibitory activity	70
euphorfinoid M **(166)**	*Euphorbia fischeriana*				71
euphorfinoid N **(167)**	*Euphorbia fischeriana*	Hela	3.62 μM	antiproliferative activity	71
difischenoid B **(168)**	*Euphorbia fischeriana*	Hela<br>(ROS, Ca2+, mitochondrial membrane potential)	3.75 μM	antiproliferative activity	35
difischenoid C **(169)**	*Euphorbia fischeriana*				35
difischenoid D **(170)**	*Euphorbia fischeriana*				35
euphohelide A **(171)**	*Euphorbia helioscopia*	RAW264.7 cells stimulated by LPS	32.98 μM	anti-inflammatory activity	72

		(NF-κB)			
euphohelide B **(172)**	*Euphorbia helioscopia*				72
euphohelide C **(173)**	*Euphorbia helioscopia*				72
zanzibariolide A **(174)**	*Suregada zanzibariensis*				73
zanzibariolide B **(175)**	*Suregada zanzibariensis*				73
17-hydroxy,11*α*, 8(14) epoxy-*ent*-abieta-13(15)-ene-11,12-dioxide **(176)**	*Euphorbia fischeriana*				74

*All references listed in the Supplementary File are included in the main text and the corresponding reference numbers in the last column are consistent with those in the main text.

Table S4 Compound names, plant sources and their reported activities of dimeric *ent*-abietane diterpenoids

Compound name	Plant sources	Cell lines/Test model	IC_{50}	Reported activities	Reference*
bisebracteolasin A **(177)**	*Euphorbia ebracteolata*	HL-60,A549,SMMC-7721,MCF,SW-480	2.61-14.09 μM	cytotoxicity activity	83
		P6C	16.48 μM		
bisebracteolasin B **(178)**	*Euphorbia ebracteolata*	HL-60,A549,SMMC-7721,MCF,SW-480	2.61-14.09 μM	cytotoxicity activity	83
		P6C	34.76 μM		
fischdiabietane A **(179)**	*Euphorbia fischeriana*	T47D	6.51 μM	antitumor activity	84
bisfischoid A **(180)**	*Euphorbia fischeriana*	sEH (Tyr343)	9.9 μM	anti-inflammatory activity	85
bisfischoid B **(181)**	*Euphorbia fischeriana*	sEH (Tyr343)	10.29 μM	anti-inflammatory activity	85
biseupyiheoid A **(182)**	*Euphorbia fischeriana*	LoVo	6.7 μM	cytotoxicity activity	86
bisfischoid C **(183)**	*Euphorbia fischeriana*				86
bislangduoid A **(184)**	*Euphorbia fischeriana*	HepG2	7.4 μM	cytotoxicity activity	87
bislangduoid B **(185)**	*Euphorbia fischeriana*				87
biseuphoid A **(186**)	*Euphorbia fischeriana*	sEH	8.17 μM	anti-inflammatory activity	88
biseuphoid B **(187)**	*Euphorbia fischeriana*	sEH	5.61 μM	anti-inflammatory activity	88

*All references listed in the Supplementary File are included in the main text and the corresponding reference numbers in the last column are consistent with those in

the main text.

Table S5 Compound names, plant sources and their reported activities of miscellaneous *ent*-abietane diterpenoids

Compound name	Plant sources	Cell lines/Test model	IC_{50}	Reported activities	Reference*
chlorabietin A **(188)**	*Chloranthus oldhamii*				22
chlorabietin B **(189)**	*Chloranthus oldhamii*	LPS-activated murine BV-2 microglial cells	16.4-33.8 μM	anti-inflammatory activity	22
chlorabietin C **(190)**	*Chloranthus oldhamii*	LPS-activated murine BV-2 microglial cells	16.4-33.8 μM	anti-inflammatory activity	22
chlorabietin D **(191)**	*Chloranthus oldhamii*				22
chlorabietin E **(192)**	*Chloranthus oldhamii*				22
chlorabietin F **(193)**	*Chloranthus oldhamii*	LPS-activated murine BV-2 microglial cells	16.4-33.8 μM	anti-inflammatory activity	22
phorneroid A **(194)**	*Euphorbia neriifolia*	HL-60	9.9 μM	cytotoxicity activity	61
decandrol A **(195)**	*Ceriops decandra*				26
fischeriana A **(196)**	*Euphorbia fischeriana*	HepG2	15.75 μM	cytotoxicity activity	89
euphoractone **(197)**	*Euphorbia fischeriana*	H23	21.07μM	cytotoxicity activity	90
		H460	20.91 μM		

*All references listed in the Supplementary File are included in the main text and the corresponding reference numbers in the last column are consistent with those in the main text.

References

21. Kuang, X.; Li, W.; Kanno, Y.; Yamashita, N.; Kikkawa, S.; Azumaya, I.; Nemoto, K.; Asada, Y.; Koike, K. Euphorins A-H: bioactive diterpenoids from *Euphorbia fischeriana*. *J. Nat. Med.* **2016**, *70*, 412-422.
22. Xiong, J.; Hong, Z.-L.; Xu, P.; Zou, Y.; Yu, S.-B.; Yang, G.-X.; Hu, J.-F. *ent*-Abietane diterpenoids with anti-neuroinflammatory activity from the rare Chloranthaceae plant *Chloranthus oldhamii*. *Org. Biomol. Chem.* **2016**, *14*, 4678-4689.
23. Yang, J.; An, Y.; Wu, H.; Liu, M.; Wang, W.; Du, X.; Li, Y.; Pu, J.; Sun, H. *Ent*-kaurane and *ent*-abietane diterpenoids from *Isodon phyllostachys*. *Sci. China: Chem.* **2016**, *59*, 1211-1215.
24. Wan, J.; Jiang, H.-Y.; Tang, J.-W.; Li, X.-R.; Du, X.; Li, Y.; Sun, H.-D.; Pu, J.-X. *Ent*-abietanoids isolated from *Isodon serra*. *Molecules* **2017**, *22*, 309.
25. Zhang, J.; He, J.; Wang, X.-X.; Shi, Y.-X.; Zhang, N.; Ma, B.-Z.; Zhang, W.-K.; Xu, J.-K. *Ent*-abietane diterpenoids and their probable biogenetic precursors from the roots of *Euphorbia fischeriana*. *RSC Adv.* **2017**, *7*, 55859-55865.
26. Jiang, Z.-P.; Tian, L.-W.; Shen, L.; Wu, J. *Ent*-abietanes from the *Godavari mangrove*, *Ceriops decandra*: absolute configuration and NF-κB inhibitory activity. *Fitoterapia* **2018**, *130*, 272-280.
27. Liu, G.L.; Xu, W.; Liu, X.J.; Yan, X.L.; Chen, J. Two new abietane diterpenoids from the leaves of *Rabdosia serra*. *J. Asian Nat. Prod. Res.* **2020**, *22*, 47-51.
28. Chen, L.; Yang, Q.; Hu, K.; Li, X.-N.; Sun, H.-D.; Puno, P.-T. Isoforrethins A–D, four *ent*-abietane diterpenoids from *Isodon forrestii* var. *forrestii*. *Fitoterapia* **2019**, *134*, 158-164.
29. Liu, J.-L.; Yu, M.; Liao, H.-B.; Liu, T.; Tan, Y.-H.; Liang, D.; Zhang, G.-J. Sesquiterpenes and diterpenes from *Euphorbia thymifolia*. *Fitoterapia* **2019**, *139*, 104408.
30. Yan, X.-L.; Zhang, J.-S.; Huang, J.-L.; Zhang, Y.; Chen, J.-Q.; Tang, G.-H.; Yin, S. Euphonoids A−G, cytotoxic diterpenoids from *Euphorbia fischeriana*. *Phytochemistry* **2019**, *166*, 112064.
31. Li, D.-W.; Deng, X.-P.; He, X.; Han, X.-Y.; Ma, Y.-F.; Huang, H.-L.; Yu, Z.-L.; Feng, L.; Wang, C.; Ma, X.-C. Eupholides A−H, abietane diterpenoids from the roots of *Euphorbia fischeriana*, and their bioactivities. *Phytochemistry* **2021**, *183*, 112593.
32. Isyaka, S.M.; Langat, M.K.; Mas-Claret, E.; Mbala, B.M.; Mvingu, B.K.; Mulholland, D.A. *Ent*-abietane and *ent*-pimarane diterpenoids from *Croton mubango* (Euphorbiaceae). *Phytochemistry* **2020**, *170*, 112217.
34. Yan, X.-L.; Huang, J.-L.; Tang, Y.-Q.; Tang, G.-H.; Yin, S. Euphopanes A–C, three new diterpenoids from *Euphorbia pekinensis*. *Nat. Prod. Res.* **2020**, *36*, 114-121.
35. Wei, J.C.; Gao, Y.N.; Wang, D.D.; Zhang, X.Y.; Fan, S.P.; Bao, T.R.G.; Gao, X.X.; Hu, G.S.; Wang, A.H.; Jia, J.M. Discovery of highly oxidized abietane diterpenoids from the roots of *Euphorbia fischeriana* with anti - tumor activities. *Chin. J. Chem.* **2021**, *39*, 2973-2982.
36. Wang, W.; Dong, L.-B. Antimicrobial *ent*-abietane diterpenoids from the leaves of *Croton cascarilloide*. *J. Asian Nat. Prod. Res.* **2022**, *25*, 68-74.
37. Wang, W.; Zhang, X.-J. Cytotoxic *ent*-abietane diterpenoids from the leaves of *Croton lachnocarpus* Benth. *J. Asian Nat. Prod. Res.* **2022**, *25*, 309-315.
38. Zhu, Q.-F.; Xu, G.-B.; Liao, S.-G.; Yan, X.-L. *Ent*-abietane diterpenoids from *euphorbia fischeriana* and their cytotoxic activities. *Molecules* **2022**, *27*, 7258.
39. Xia, J.-N.; Hu, K.; Su, X.-Z.; Tang, J.-W.; Li, X.-N.; Sun, H.-D.; Puno, P.-T. Discovery of *ent*-kaurane diterpenoids, characteristic metabolites of *Isodon* species, from an endophytic fungal strain *Geopyxis* sp. XY93 inhabiting *Isodon parvifolia*. *Fitoterapia* **2022**, *158*, 105160.
40. Zhu, H.; Wang, J.; Hu, W.; Zhou, T.; Lin, Z.; Zhang, R.; Geng, C.-a.; Chen, X. Diterpenoids with cytotoxicity for pancreatic cancer SW1990 cells from the rhizomes of *Euphorbia jolkinii* boiss. *Chin. J. Org. Chem.* **2024**, *44*, 1929-1937.
41. Zhang, H.-L.; Zhang, Y.; Yan, X.-L.; Xiao, L.-G.; Hu, D.-X.; Yu, Q.; An, L.-K. Secondary metabolites from *Isodon ternifolius* (D. Don) Kudo and their anticancer activity as DNA topoisomerase IB and Tyrosyl-DNA phosphodiesterase 1 inhibitors. *Bioorganic Med. Chem.* **2020**, *28*, 115527.
42. Tang, X.; Xu, J.-L.; Li, X.-Y.; Zhang, Y.-Y.; Xiang, S.-Q.; Luo, X.; Liu, Z.-Q.; Meng, X.-L.; Zhou, H.; Wu, P. Diterpenoids with anti-inflammatory activities from *Isodon rubescens*. *Fitoterapia* **2025**, *185*, 106759.

43. Yun, Y.S.; Shimamura, M.; Fukaya, H.; Fuchino, H.; Kawahara, N.; Inoue, H. Lathyrisol B, a new nor-*ent*-abietane diterpenoid from roots of *Euphorbia lathyris* L. *Phytochem. Lett.* **2025**, *65*, 113-116.
44. Zhang, M.-W.; Guo, K.; Zhang, Y.; Teng, L.-L.; Huang, Q.-P.; Liu, Y.; Li, S.-H. Leucoabietenes A and B, rearranged abietane and *ent*-abietane diterpene hydrocarbons against resistant infectious fungus and bacterium from the leaves of *Leucosceptrum canum*. *Tetrahedron Lett.* **2021**, *81*, 153356.
45. Essa, A.F.; El-Hawary, S.S.; Emam, S.E.; Kubacy, T.M.; El-Khrisy, E.E.-D.A.M.; Younis, I.Y.; Elshamy, A.I. Characterization of undescribed melanoma inhibitors from *Euphorbia mauritanica* L. cultivated in Egypt targeting BRAFV600E and MEK 1 kinases via *in-silico* study and ADME prediction. *Phytochemistry* **2022**, *198*, 113154.
46. Li, H.-Y.; Bao, M.-Y.; Xiong, H.-M.; Wang, C.-C.; Bai, L.-P.; Zhang, W.; Chen, C.-Y.; Jiang, Z.-H.; Zhu, G.-Y. Forsyditerpenes A–O, CC-type clerodane and aromatic abietane diterpenoids with anti-inflammatory activities from the seeds of *Forsythia suspensa*. *Fitoterapia* **2025**, *185*, 106675.
47. Li, J.; Meng, X.; Yin, C.; Zhang, L.; Lin, B.; Liu, P.; Zhu, L.; Wang, H.; Liu, H.; Zhang, X.; et al. Antimalarial and neuroprotective *ent*-abietane diterpenoids from the aerial parts of *Phlogacanthus curviflorus*. *Chin J Nat Med* **2023**, *21*, 619-630.
48. Liu, Z.-g.; Li, Z.-l.; Li, D.-h.; Li, N.; Bai, J.; Zhao, F.; Meng, D.-l.; Hua, H.-m. *Ent*-abietane-type diterpenoids from the roots of *Euphorbia ebracteolata* with their inhibitory activities on LPS-induced NO production in RAW 264.7 macrophages. *Bioorg. Med. Chem. Lett.* **2016**, *26*, 1-5.
49. Wang, C.-J.; Yan, Q.-L.; Ma, Y.-F.; Sun, C.-P.; Chen, C.-M.; Tian, X.-G.; Han, X.-Y.; Wang, C.; Deng, S.; Ma, X.-C. *Ent*-abietane and tigliane diterpenoids from the roots of *Euphorbia fischeriana* and their inhibitory effects against *Mycobacterium smegmatis*. *J. Nat. Prod.* **2017**, *80*, 1248-1254.
50. Han, C.; Peng, Y.; Wang, Y.; Huo, X.; Zhang, B.; Li, D.; Leng, A.; Zhang, H.; Ma, X.; Wang, C. Cytotoxic *ent*-abietane-type diterpenoids from the roots of *Euphorbia ebracteolata*. *Bioorg. Chem.* **2018**, *81*, 93-97.
51. Ma, Y.-L.; Tang, X.-H.; Yuan, W.-J.; Ding, X.; Di, Y.-T.; Hao, X.-J. Abietane diterpernoids from the roots of *euphorbia ebracteolata*. *Nat. Prod. Bioprospect.* **2018**, *8*, 131-135.
52. Mangisa, M.; Tembu, V.J.; Fouche, G.; Nthambeleni, R.; Peter, X.; Langat, M.K. *Ent*-abietane diterpenoids from *Suregada zanzibariensis* Baill. (Euphorbiaceae), their cytotoxic and anticancer properties. *Nat. Prod. Res.* **2018**, *33*, 3240-3247.
53. Li, J.-C.; Zhang, Z.-J.; Yang, T.; Jiang, M.-Y.; Liu, D.; Li, H.-M.; Li, R.-T. Six new *ent*-abietane-type diterpenoids from the stem bark of *Euphorbia neriifolia*. *Phytochem. Lett.* **2019**, *34*, 13-17.
54. Wang, P.; Xie, C.; An, L.; Yang, X.; Xi, Y.; Yuan, S.; Zhang, C.; Tuerhong, M.; Jin, D.-Q.; Lee, D.; et al. Bioactive diterpenoids from the stems of *Euphorbia royleana*. *J. Nat. Prod.* **2019**, *82*, 183-193.
55. Yin, Z.; Xie, X.-L.; Yuan, J.; Zhang, Y.; Li, W. Two new *ent*-abietane diterpenoids from *Euphorbia helioscopia*. *J. Asian Nat. Prod. Res.* **2019**, *22*, 632-638.
56. Chen, Y.-N.; Lu, Q.-Y.; Li, D.-M.; Li, Y.-Y.; Pu, X.-X.; Li, B.-T.; Tang, X.-H.; Tang, H.-Y.; Liu, S.; Yang, L.; et al. Three new diterpenoids from *Euphorbia peplus*. *Nat. Prod. Res.* **2020**, *35*, 3901-3907.
57. Silva, M.L.; Costa-Silva, T.A.; Antar, G.M.; Tempone, A.G.; Lago, J.H.G. Chemical constituents from aerial parts of *Baccharis sphenophylla* and effects against intracellular forms of *Trypanosoma cruzi*. *Chem. Biodiversity* **2021**, *18*, e2100466.
58. Li, H.; Yang, P.; Zhang, E.-H.; Kong, L.-M.; Meng, C.-Y. Antimicrobial *ent*-abietane-type diterpenoids from the roots of *Euphorbia wallichii*. *J. Asian Nat. Prod. Res.* **2020**, *23*, 652-659.
59. Yuan, W.-J.; Gao, W.-F.; Zhao, J.-Y.; Zhang, Y.; Chen, D.-Z.; Li, S.-L.; Di, Y.-T.; Hao, X.-J. Diterpenes with potential treatment of vitiligo from the aerials parts of *Euphorbia antiquorum* L. *Fitoterapia* **2020**, *144*, 104583.
60. Chokchaisiri, S.; Apiratikul, N.; Rukachaisirikul, T. A new *ent*-abietane lactone from *Glycosmis pentaphylla*. *Nat. Prod. Res.* **2020**, *34*, 3019-3026.

61. Gao, Y.; Zhou, J.-S.; Liu, H.-C.; Zhang, Y.; Yin, W.-H.; Liu, Q.-F.; Wang, G.-W.; Zhao, J.-X.; Yue, J.-M. Phorneroids A–M, diverse types of diterpenoids from *Euphorbia neriifolia*. *Phytochemistry* **2022**, *198*, 113142.
62. Ran, X.; Lu, Q.-Y.; Li, Y.-Y.; Pu, X.-X.; Guo, Y.; Yuan, M.-R.; Guan, S.-P.; Sun, M.; Jiao, L.; Yao, Y.-G.; et al. Euphejolkinolide A, a new *ent*-abietane lactone from *Euphorbia peplus* L. with promising biological activity in activating the autophagy-lysosomal pathway. *Heliyon* **2023**, *9*, e13691.
63. Matundura, J.O.; Mollel, J.T.; Miah, M.; Said, J.; Omosa, L.K.; Kalenga, T.M.; Woordes, Y.T.; Nchiozem-Ngnitedem, V.-A.; Orthaber, A.; Midiwo, J.O.; et al. Bioactive abietenolide diterpenes from *Suregada procera*. *Fitoterapia* **2024**, *179*, 106217.
64. Fu, X.; Yu, D.; Zhu, G.; Xu, J. Three new abietane diterpenoids from the aerial parts of *Euphorbia fischeriana* and their cytotoxic effects. *Phytochem. Lett.* **2023**, *55*, 56-60.
65. Olaranont, Y.; Mas-Claret, E.; Cheek, M.; Prescott, T.A.K.; Onana, J.M.; Langat, M.K. Cytotoxic *ent*-abietane diterpenoids, banyangmbolides A-E, from the leaves of *Suregada occidentalis*. *Heliyon* **2024**, *10*, e25917.
66. Shakeri, A.; Mirahmadi, M.R.; Kunert, O.; Tsai, Y.-C.; Barta, A.; Hohmann, J.; Asili, J. Diverse diterpenoids and a triterpenoid from *Euphorbia spinidens* Bornm. ex Prokh. *Fitoterapia* **2024**, *173*, 105838.
67. Yang, H.-Y.; Huang, P.-Z.; Feng, W.-J.; Si, P.-W.; Gao, K.; Chen, J.-J. *ent*-Abietane-type lactones with anti-inflammatory activity from *Euphorbia helioscopia*. *Phytochemistry* **2025**, *229*, 114313.
68. Yan, Y.; Peng, M.-Y.; Yang, Y.; Zhang, Z.-B.; Zhang, L.-L.; Tang, L.; Qin, X.-J.; Cheng, Y.-Y.; Di, Y.-T.; Hao, X.-J. Highly oxygenated *ent*-abietane diterpenoid lactones from *Euphorbia peplus* and their anti-inflammatory activity. *Bioorg. Chem.* **2025**, *154*, 107989.
69. Wang, W.-P.; Jiang, K.; Zhang, P.; Shen, K.-K.; Qu, S.-J.; Yu, X.-P.; Tan, C.-H. Highly oxygenated and structurally diverse diterpenoids from *Euphorbia helioscopia*. *Phytochemistry* **2018**, *145*, 93-102.
70. Wei, J.-C.; Zhang, X.-Y.; Gao, Y.-N.; Wang, D.-D.; He, X.-L.; Gao, X.-X.; Hu, G.-S.; Wang, A.-H.; Jia, J.-M. Euphorfinoids E-L: diterpenoids from the roots of *Euphorbia fischeriana* with acetylcholinesterase inhibitory activity. *Phytochemistry* **2021**, *190*, 112867.
71. Zhao, Y.; Huang, H.-H.; Wei, J.-C.; Wang, Q.; Long, G.-Q.; Wang, A.-H.; Jia, J.-M. Antiproliferative *ent*-abietane diterpenoids from *Euphorbia fischeriana*. *Nat. Prod. Res.* **2023**, *37*, 4081-4088.
72. Yang, H.-Y.; Yao, W.; Huang, P.-Z.; Xu, H.; Ma, Q.; Chen, X.; Chen, J.-J.; Gao, K. Euphohelides A–C, *ent*-abietane-type norditerpene lactones from *Euphorbia helioscopia* and their anti-inflammatory activities. *J. Nat. Prod.* **2023**, *86*, 1003-1009.
73. Kalenga, T.M.; Mollel, J.T.; Said, J.; Orthaber, A.; Ward, J.S.; Atilaw, Y.; Umereweneza, D.; Ndoile, M.M.; Munissi, J.J.E.; Rissanen, K.; et al. Modified *ent*-abietane diterpenoids from the leaves of *Suregada zanzibariensis*. *J. Nat. Prod.* **2022**, *85*, 2135-2141.
74. Adelakun, T.A.; Ding, X.; Ombati, R.M.; Zhao, N.-D.; Obodozie-Ofoegbu, O.O.; Di, Y.-T.; Zhang, Y.; Hao, X.-J. A new highly oxygenated abietane diterpenoid and a new lysosome generating phorbol ester from the roots of *Euphorbia fischeriana* Steud. *Nat. Prod. Res.* **2019**, *34*, 3027-3035.
83. Yuan, W.-J.; Ding, X.; Wang, Z.; Yang, B.-J.; Li, X.-N.; Zhang, Y.; Chen, D.-Z.; Li, S.-L.; Chen, Q.; Di, Y.-T.; et al. Two novel diterpenoid heterodimers, bisebracteolasins A and B, from *Euphorbia ebracteolata* Hayata, and the cancer chemotherapeutic potential of bisebracteolasin A. *Sci. Rep.* **2017**, *7*, 1-9.
84. He, J.; Xu, J.-K.; Guo, L.-B.; Xia, C.-Y.; Lian, W.-W.; Tian, H.-Y.; Zhang, J.; Shi, Y.-X.; Zhang, W.-K. Fischdiabietane A, an antitumoral diterpenoid dimer featuring an unprecedented carbon skeleton from *Euphorbia fischeriana*. *J. Org. Chem.* **2021**, *86*, 5894-5900.
85. Sun, C.-P.; Chang, Y.-B.; Wang, C.; Lv, X.; Zhou, W.-Y.; Tian, X.-G.; Zhao, W.-Y.; Ma, X.-C. Bisfischoids A and B, dimeric *ent*-abietane-type diterpenoids with anti-inflammatory potential from *Euphorbia fischeriana* Steud. *Bioorg. Chem.* **2021**, *116*, 105356.
86. Peng, Y.; Chang, Y.; Sun, C.; Wang, W.; Wang, C.; Tian, Y.; Zhang, B.; Deng, S.; Zhao, W.; Ma, X. Octacyclic and decacyclic *ent*-abietane dimers with cytotoxic activity from *Euphorbia fischeriana* steud. *Chin. Chem. Lett.* **2022**, *33*, 4261-4263.

87. Yu, Z.-L.; Zhou, M.-R.; Wang, W.-Y.; Chang, Y.-B.; Sun, C.-P.; Lv, X.; Wang, C.; Zhao, W.-Y.; Ma, X.-C. Cytotoxic diterpenoid dimer containing an intricately caged core from *Euphorbia fischeriana*. *Bioorg. Chem.* **2022**, *123*.
88. Zhao, W.-Y.; Sun, C.-P.; Chang, Y.-B.; Wang, W.-Y.; Yan, J.-K.; Lv, X.; Wang, C.; Ma, X.-C. Unprecedented diterpenoid dimers with soluble epoxide hydrolase inhibitory effect from *Euphorbia fischeriana*. *Org. Biomol. Chem.* **2022**, *20*, 2508-2517.
89. He, J.; Xu, J.-K.; Zhang, J.; Bai, H.-J.; Ma, B.-Z.; Cheng, Y.-C.; Zhang, W.-K. Fischeriana A, a meroterpenoid with an unusual 6/6/5/5/5/6/6 heptacyclic carbon skeleton from the roots of *Euphorbia fischeriana*. *Org. Biomol. Chem.* **2019**, *17*, 2721-2724.
90. Xie, R.; Li, L.; Fan, X.; Zi, J. Euphoractone, a cytotoxic meroterpenoid with an unusual *ent*-abietane-phloroglucinol skeleton, from *Euphorbia fischeriana* Steud. *Chin. Chem. Lett.* **2020**, *31*, 431-433.
